# A stepwise model of reaction-diffusion and positional information governs self-organized human peri-gastrulation-like patterning

**DOI:** 10.1242/dev.149658

**Published:** 2017-12-01

**Authors:** Mukul Tewary, Joel Ostblom, Laura Prochazka, Teresa Zulueta-Coarasa, Nika Shakiba, Rodrigo Fernandez-Gonzalez, Peter W. Zandstra

**Affiliations:** 1 Institute of Biomaterials and Biomedical Engineering (IBBME), University of Toronto, Toronto, Ontario, M5S 3E1, Canada; 2 Collaborative Program in Developmental Biology, University of Toronto, Toronto, Ontario, M5S 3E1, Canada; 3 Terrence Donnelly Centre for Cellular and Biomolecular Research, University of Toronto, Toronto, Ontario, M5S 3E1, Canada; 4 Ted Rogers Centre for Heart Research, University of Toronto, Toronto, Ontario, M5G 1M1, Canada; 5 Department of Cell and Systems Biology, University of Toronto, Toronto, Ontario, M5S 3G5, Canada; 6 Department of Chemical Engineering and Applied Chemistry, University of Toronto, Toronto, Ontario, M5S 3ES, Canada; 7 Medicine by Design: A Canada First Research Excellence Fund Program, University of Toronto, Toronto, Ontario, M5S 3E1, Canada

**Keywords:** Developmental organoids, Human gastrulation, Morphogenesis, Pluripotent stem cells, Positional information, Reaction-diffusion

## Abstract

How position-dependent cell fate acquisition occurs during embryogenesis is a central question in developmental biology. To study this process, we developed a defined, high-throughput assay to induce peri-gastrulation-associated patterning in geometrically confined human pluripotent stem cell (hPSC) colonies. We observed that, upon BMP4 treatment, phosphorylated SMAD1 (pSMAD1) activity in the colonies organized into a radial gradient. We developed a reaction-diffusion (RD)-based computational model and observed that the self-organization of pSMAD1 signaling was consistent with the RD principle. Consequent fate acquisition occurred as a function of both pSMAD1 signaling strength and duration of induction, consistent with the positional-information (PI) paradigm. We propose that the self-organized peri-gastrulation-like fate patterning in BMP4-treated geometrically confined hPSC colonies arises via a stepwise model of RD followed by PI. This two-step model predicted experimental responses to perturbations of key parameters such as colony size and BMP4 dose. Furthermore, it also predicted experimental conditions that resulted in RD-like periodic patterning in large hPSC colonies, and rescued peri-gastrulation-like patterning in colony sizes previously thought to be reticent to this behavior.

## Abstract (precis)

**Summary:** A high-throughput *in vitro* system allowing the induction of peri-gastrulation-like fates in geometrically confined hPSC colonies reveals that a two-step process underlies the observed self-organization and subsequent fate acquisition.

## INTRODUCTION

During development, pluripotent stem cells (PSCs) in the epiblast are exposed to signaling gradients that initiate a sequence of fate specifications and cell movements resulting in spatial segregation of the germ layers in a developmentally conserved process called gastrulation (Rossant and Tam, 2004, 2009; Morris et al., 2012; Tam et al., 2006; Ciruna and Rossant, 2001). A number of model organisms have been used to study the molecular mechanisms that underpin the fate patterning that arises during this crucial developmental checkpoint (Solnica-Krezel and Sepich, 2012; Leptin, 2005; Keller, 2005; Nakaya and Sheng, 2008; Tam and Behringer, 1997; Tam and Loebel, 2007; Myers et al., 2002). In all model organisms studied, transforming growth factor beta (TGFβ) superfamily members, including bone morphogenetic proteins (BMPs) and Nodal, which signal though a family of mediator proteins called SMADs, play key roles in inducing gastrulation-specific pattern formation (Wu and Hill, 2009). Although involvement of the TGFβ superfamily members is conserved between species during gastrulation, details of fate patterning in relation to cell movements, tissue structure, specific molecules involved, etc., can vary between species (Wu and Hill, 2009; Rossant, 2015; Narasimha and Leptin, 2000; Solnica-Krezel and Sepich, 2012; Solnica-Krezel, 2005). Therefore, it has been challenging to relate the regulatory mechanisms of pattern formation in previously studied developmental models to human gastrulation.

Because human embryos are not typically available for direct investigation, studying human gastrulation-associated fate patterning requires *in vitro* platforms that allow robust simulation and investigation of the signaling programs that initiate and drive gastrulation-like events. We and others have previously used micro-patterning technologies to control human (h)PSC colony geometry, demonstrating improved cell response consistency than is achieved in conventional adherent cultures (Nazareth et al., 2013; Peerani et al., 2007; Bauwens et al., 2008, 2011; Alom Ruiz and Chen, 2007; Stevens et al., 2013; Ma et al., 2015; Rahman et al., 2017). These studies highlight that control of endogenous signaling profiles, cell-cell contact, and mechanical forces are crucial to regulate cell fate and spatial tissue organization robustly. Recently, Warmflash et al. (2014) used similar techniques to demonstrate that following BMP4 treatment geometrically controlled hPSC colonies recapitulate many aspects of the peri-gastrulation-stage epiblast. Specifically, these colonies exhibited spatially patterned regions characteristic of primitive streak-like, mesoderm-like, endoderm-like, ectoderm-like and trophoblast-like tissues (Warmflash et al., 2014).

Two prominent biochemical models have influenced our understanding of the cell fate patterning and morphogenesis that occurs during embryogenesis. The first is reaction-diffusion (RD), which describes the self-organization of homogenously distributed signaling molecules (morphogens) into complex, asymmetric patterns that provide spatial information to developing tissues (Turing, 1952; Gierer and Meinhardt, 1972). The second is positional information (PI), which describes how the asymmetric morphogen distributions across a developing tissue can be interpreted, and result in cell fate patterning (Wolpert, 1981, 1969). RD hypothesizes the presence of an interaction network of two molecules: an ‘activator’, which activates the expression of both molecules, and an ‘inhibitor’, which inhibits their expression. This interaction network, in conjunction with dissimilar activator and inhibitor diffusivities, is theoretically sufficient to self-organize asymmetrical morphogen distributions (Turing, 1952; Gierer and Meinhardt, 1972). The initial version of PI proposed a mechanism by which this asymmetric morphogen distribution could be translated into patterned developmental fates through a signaling threshold-based mechanism. Subsequent studies using the developing neural tube have demonstrated that fate patterning by PI is not just mediated by morphogen threshold levels, but is a function of both the morphogen concentration and exposure duration (Dessaud et al., 2007, 2008; Briscoe and Small, 2015).

Here, we demonstrate, using fully defined and scalable conditions, that geometrically confined hPSC colonies organize into radially segregated regions that express markers characteristic of ectoderm-like, primitive streak-like and trophoblast-like tissues. We show that upon BMP4 induction, an RD network regulated by BMP4 and noggin (NOG; a cardinal BMP inhibitor) rapidly organizes nuclear localized phosphorylated (p)SMAD1 (an effector of BMP signaling) into a gradient within the geometrically confined colonies. The established gradient then patterns hPSC differentiation in a manner consistent with PI. We developed a computational model of a BMP4-NOG RD system and demonstrate that, across a range of colony sizes and BMP4 doses, RD consistently predicts the formation of pSMAD1 signaling gradient and PI accurately predicts the patterned fate acquisition. A stepwise model of RD-mediated self-organization of a pSMAD1 signaling gradient followed by a PI-mediated patterning of cell fates can predict the outcome of previously unexplored experimental conditions. Specifically, we both predict and observe periodic fate patterning consistent with the cardinal RD paradigm. Furthermore, our model can identify conditions that rescue fate patterning in colonies previously deemed incapable of facilitating pattern formation (Warmflash et al., 2014). Taken together, our data support the concept that a stepwise process of RD and PI controls the peri-gastrulation-like patterns that develop in differentiating hPSC colonies.

## RESULTS

### A defined high-throughput assay for induction of peri-gastrulation-like patterning in human pluripotent stem cell colonies

**Fig. 1.**
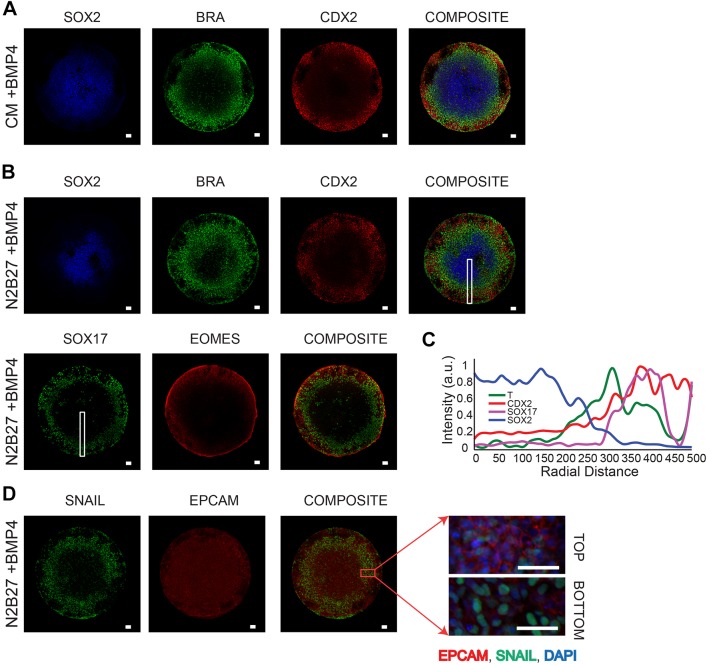
**Defined peri-gastrulation-like patterning** **induction in differentiating hPSC colonies.** (A,B) Representative immunofluorescence images of fate patterning of SOX2, BRA and CDX2 in BMP4-supplemented CM (A) as previously reported (Warmflash et al., 2014; Etoc et al., 2016), and fate patterning in BMP4-supplemented N2B27 medium stained for SOX2, BRA, CDX2, SOX17 and EOMES (B). (C) Spatial trends for intensity of expression of SOX2, BRA (T), CDX2 and SOX17 in regions marked by white rectangles in B and C (average trends of replicates shown in Figs S1 and S2). (D) Representative images of SNAIL and EPCAM staining in a micro-patterned colony differentiated in N2B27 shows that mesenchymal marker-expressing cells in the primitive streak region are located underneath an epithelial layer. Scale bars: 50 µm.

Consistent with recent reports (Warmflash et al., 2014; Etoc et al., 2016), we observed radially segregated expression of the gastrulation-associated markers CDX2, brachyury (T; abbreviated here as BRA) and SOX2 representative of trophoblast-like, primitive streak-like and ectoderm-like tissues, respectively – develop in geometrically confined circular hPSC colonies differentiated in mouse embryonic fibroblast-conditioned medium (CM) supplemented with BMP4 (Fig. 1A). Given the challenge of identifying key molecules regulating pattern formation in undefined CM, our first aim was to identify defined basal conditions that induce these patterns in hPSC colonies. Further, we optimized a previously described protocol that uses deep ultraviolet light (<200 nm)-mediated photo-oxidation of polyethylene glycol (PEG)-coated slides (Azioune et al., 2009) to allow high-fidelity patterning of hPSC colonies and adapted this technique to produce micropatterned 96-well microtiter plates (Materials and Methods).

Using our PEG plates, we performed a medium screen to identify defined conditions to induce patterning of gastrulation-associated fates in geometrically confined hPSC colonies. The screen consisted of Nutristem (NS), mTeSR (MT), Essential-8 (E8), a KnockOut Serum Replacement-based medium (SR) and an N2B27-based medium, all supplemented with BMP4. Preliminary testing with N2B27 medium revealed that NODAL supplementation elicited a positive response in the induction of the BRA-expressing region (data not shown), consistent with the importance of Nodal signaling in the specification of the primitive streak fate (Funa et al., 2015; Schier, 2003; Brennan et al., 2001; Shen, 2007). Therefore, we performed all N2B27-based experiments with supplemented NODAL (100 ng/ml). We found that, although segregated expression of CDX2 and SOX2 developed in all defined test media, BRA expression was not consistently observed in E8, NS and SR media (Figs S1 and S2). Further, although spatial segregation of CDX2, BRA and SOX2 occurred reproducibly in MT medium, colonies patterned in MT tended to lift off the plate by 48 h. N2B27 medium robustly gave rise to differentiating hPSC colonies with regions expressing trophoblast-associated (CDX2) and primitive streak-associated (BRA) markers, with concurrent spatial patterning of expression markers indicative of endoderm-like (SOX17) and mesoderm-like (EOMES) fates, in a manner indistinguishable from hPSC colonies differentiated in BMP4-supplemented CM (Fig. 1A-C, Figs S1 and S2). Further, the regions at the center of the differentiating colonies expressed SOX2 (Fig. 1B) but not NANOG (Fig. S3), indicating a prospective ectoderm-like fate. Notably, the SNAIL (SNAI1)-expressing mesenchymal cells appeared underneath the EPCAM-expressing epithelial layer in the regions expressing markers of the primitive streak (Fig. 1D), which parallels the organization of the mesenchymal and epithelial cells at the onset of gastrulation. Also, this observation is consistent with what has previously been reported in BMP4-supplemented CM (Warmflash et al., 2014). Given these results, we define the fate patterning observed in BMP4-supplemented N2B27 medium as ‘peri-gastrulation-like’, and, unless otherwise stated, this induction medium was used for all studies detailed below.

### Nodal signaling is necessary for BRA expression but does not induce peri-gastrulation-like patterning

**Fig. 2.**
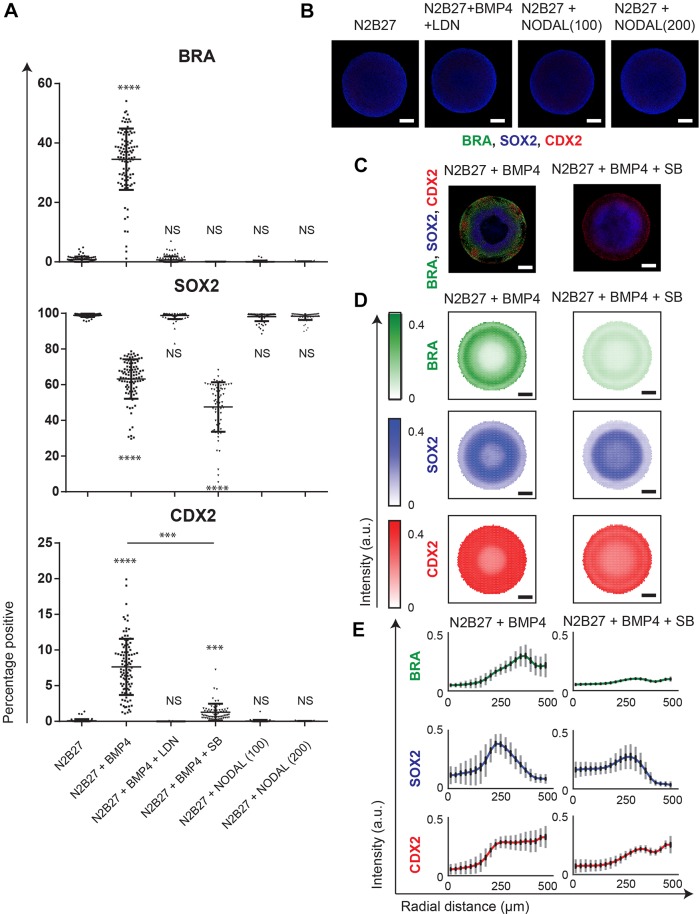
**Nodal signaling is required for primitive streak specification, but does not induce differentiation and fate patterning in geometrically confined hPSC colonies.** (A) Percentage of cells expressing BRA, SOX2 and CDX2 in N2B27 with 100 ng/ml of NODAL (*n*=73), N2B27+BMP (*n*=100), N2B27+BMP+LDN (*n*=101), N2B27+BMP+SB (*n*=67), N2B27+100 ng/ml Nodal (*n*=68) or N2B27+200 ng/ml Nodal (*n*=65). The experiment was performed twice. Data are mean±s.d. and individual data points indicate identified colonies. ****P<0.0001, NS, not significant (*P*>0.05). *P*-values were calculated using one-way ANOVA (Dunnett's post hoc test). (B) Representative images of colonies cultured in N2B27, BMP+LDN, NODAL (100) and NODAL (200) stained for BRA, SOX2 and CDX2. (C) Representative immunofluorescence images of colonies cultured in BMP4, and BMP4+SB stained for BRA, SOX2 and CDX2. (D) SOX2, BRA and CDX2 expression averages for 100 colonies cultured in BMP4, and 67 colonies cultured in BMP4+SB. (E) Average radial trends from the data shown in D for BMP4 and BMP4+SB conditions. Standard deviations shown in gray, and 95% confidence intervals in black. Scale bars: 200 µm.

Nodal signaling is important in establishing the primitive streak fate (Hong et al., 2011; Funa et al., 2015). Given that our N2B27 medium contained NODAL, we first examined whether the spatially organized differentiation observed within the geometrically confined hPSC colonies required Nodal signaling. We found that selectively inhibiting Nodal signaling with SB-431542 (SB), an inhibitor of ALK4/5/7 receptors (ACVR1B, TGFBR1 and ACVR1C) (Inman et al., 2002), in N2B27 medium supplemented with BMP4, abrogated the expression of BRA (Fig. 2A-C) and significantly reduced the expression of CDX2 (Fig. 2A). However, patterning of regions expressing SOX2 and residual CDX2 was still maintained (Fig. 2C-E). These data indicate that Nodal signaling was necessary for the expression of BRA, and at least a subset of the CDX2, but did not induce organized fate patterning in the geometrically confined hPSC colonies. We then tested the necessity of BMP signaling to induce the peri-gastrulation-like fates within the geometrically confined hPSC colonies. To test this, we queried if the patterned fates within the hPSC colonies would arise in either N2B27 medium without supplemented BMP4, or in N2B27+BMP4 with supplementation of the small molecule BMP inhibitor LDN-193189 (LDN) (Cuny et al., 2008). We found that in both cases there was no observed peri-gastrulation-like patterning (Fig. 2A,B). Furthermore, this patterning deficiency in N2B27 without supplemented BMP4 could not be rescued by further addition of either 100 ng/ml or 200 ng/ml of NODAL (Fig. 2A,B). These data indicated that activation of BMP signaling was necessary to induce peri-gastrulation-like differentiation within the geometrically confined hPSC colonies. Taken together, these data indicate that induction of peri-gastrulation-like fate patterning requires BMP signaling and the formation of a BRA-expression region further requires Nodal activity.

### BMP4-NOG interaction network regulates pSMAD1 gradient self-organization

**Fig. 3.**
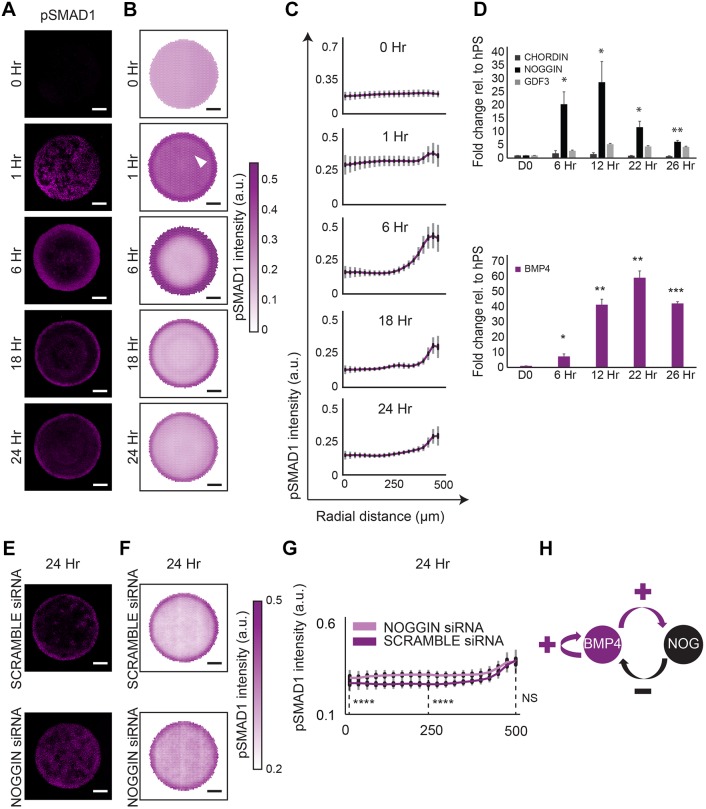
**pSMAD1 gradient self-organization in differentiating colonies suggests the presence of a BMP4**-**NOG RD network.** (A) Representative immunofluorescence images of colonies stained for pSMAD1 at 0, 1, 6, 18 and 24 h after BMP induction. (B) Average pSMAD1 intensity represented as overlays for 202, 193, 105, 100, 105 colonies for the respective induction times. Data were collected from two experiments. White arrowhead marks the region of relatively lower average pSMAD1 activation. (C) The average radial trends of pSMAD1 activity at each induction duration. Standard deviations are shown in gray, and 95% confidence intervals in black. (D) Temporal gene expression profiles for BMP signaling inhibitors (*CHRD*, *NOG* and *GDF3*) and *BMP4* at 6, 12, 22 and 26 h. Data shown as mean and s.d. of three independent experiments. *P<0.05, **P<0.01, ***P<0.001. (E) Representative pSMAD1 immunofluorescence images for colonies treated with SCRAMBLE siRNA and NOG siRNA after 24 h of BMP4 treatment. (F) Average pSMAD1 expression in colonies treated with SCRAMBLE siRNA, NOG siRNA (64 and 62 colonies, respectively). Data were collected from two experiments. (G) Radial trends of pSMAD1 signaling distribution from the data shown in F for SCRAMBLE and NOG siRNA conditions. Standard deviations are shown in gray, and 95% confidence intervals in black. ****P<0.0001, NS, not significant (*P*>0.05). *P*-values were calculated using Mann–Whitney *U*-test. (H) Overview of the implicated BMP4-NOG RD network. Scale bars: 200 µm.

Because we observed that BMP signaling was necessary for organized fate patterning within the geometrically confined hPSC colonies, we next examined how downstream effectors of BMP signaling were organized within the colonies. BMP ligands activate BMP receptors (BMPR1A, BMPR1B, BMPR2) leading to phosphorylation and nuclear translocation of SMAD1 followed by the transcription of context-specific target genes (Zhang and Li, 2005). We used an antibody specific to pSMAD1 to measure the distribution of nuclear-localized SMAD1 activity as a function of radial distance from colony centers at 1, 6, 18 and 24 h following BMP4 induction. Robust analysis was enabled by overlaying pSMAD1 expression profiles of at least 100 colonies at each time point, yielding the average intensity of pSMAD1 as a function of colony radius. We noted that 1 h after BMP4 induction, although the pSMAD1 activity appeared at all colony radii, the expression on average was slightly lower at the colony center than at the periphery (Fig. 3A), suggesting that centers of hPSC colonies might contain BMP inhibitors. This is consistent with previous reports that in the pluripotent state, hPSCs express secreted factors such as FST, CER1, GDF3, etc. (Vallier et al., 2004; Yang et al., 2015; Besser, 2004). Although these factors belong to the activin/Nodal family, they are competent inhibitors of BMP signaling (Wu and Hill, 2009). We observed relatively higher expression levels of these activin/Nodal family member inhibitors of BMP signaling than the canonical BMP inhibitors such as NOG and CHRD in hPSCs during regular culture conditions (Fig. S4). The secretion of these BMP inhibitors in the geometrically confined hPSC colony would produce a spatial distribution with the center of the colonies having relatively higher concentrations of BMP inhibitors (supplementary Materials and Methods, Fig. S14). In a colony with this expression profile of non-canonical BMP inhibitors, BMP4 treatment would result in a small reduction of BMP signaling activity at the center of the colony, as we observed (Fig. 3A). Subsequently, over the first 24 h of BMP4 treatment, we observed a rapid downregulation of pSMAD1 activity at the colony centers, and spontaneous organization into a radial gradient with the cells in the periphery exhibiting higher levels of pSMAD1 activity (Fig. 3A-C). To test whether the self-organization of pSMAD1 activity could be attributed to regulatory feedback of the BMP pathway, we measured the expression of both positive- and negative-feedback mediators of BMP signaling over the first 26 h after BMP4 treatment. We found significant upregulation in the expression of both NOG and BMP4 (Fig. 3D) in a dose-dependent manner (Fig. S5). Notably, BMP4 and NOG upregulation was seen in all basal medium conditions tested (Fig. S6), indicating that the upregulation was a response to the activation of BMP signaling and unrelated to the N2B27 medium. Furthermore, NOG knockdown, using siRNA, significantly increased pSMAD1 activity in the central regions of the geometrically confined colonies demonstrating a role for NOG in the self-organization of the pSMAD1 gradient (Fig. 3E-G, Fig. S7), as has recently been proposed by others (Etoc et al., 2016). Interestingly, along with the NOG-mediated negative feedback, we noted the presence of a positive-feedback loop in BMP signaling wherein BMP4 supplementation resulted in BMP4 expression (Fig. 3D, Fig. S6). Together, the presence of both a positive- and negative-feedback response mediated by BMP4 suggested the presence of a BMP4-NOG RD network underlying the pSMAD1 radial self-organization in differentiating hPSC colonies (Fig. 3H). We next set out to investigate properties of the RD framework as they relate to the peri-gastrulation-like patterning.

### pSMAD1 gradient formation is colony size and BMP4 concentration dependent

**Fig. 4.**
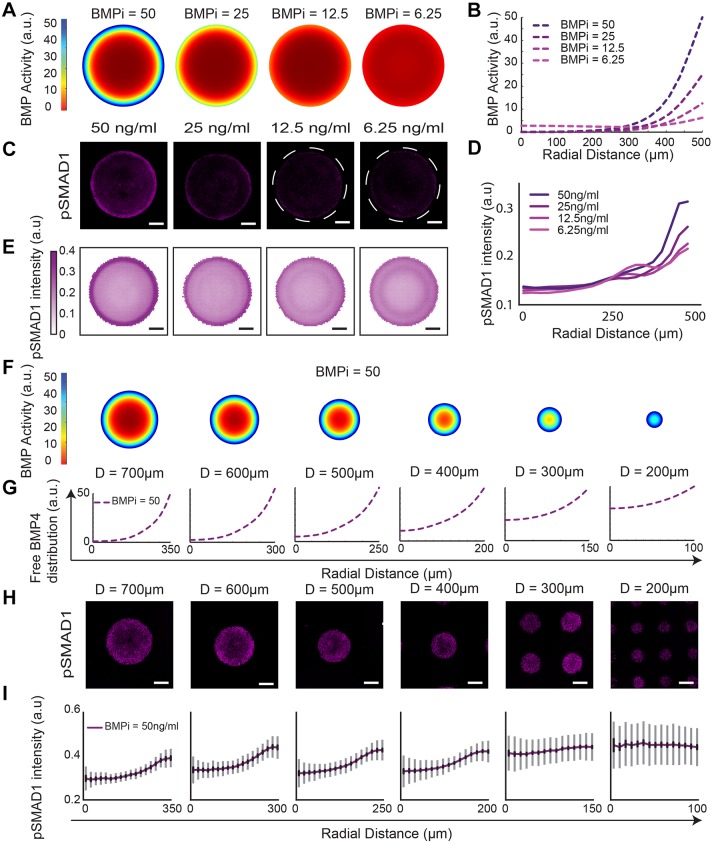
**BMP4-NOG RD model predicts pSMAD1 gradient response to colony size and BMP4 dose perturbations.** (A) Predicted distribution of free BMP4 ligands in colonies of 1000 µm diameter as a function of varying BMPi concentration (50 ng/ml, 25 ng/ml, 12.5 ng/ml and 6.25 ng/ml). (B) Line plots of predicted distributions. (C) Representative immunofluorescence images of micro-patterned colonies stained for pSMAD1 24 h after induction with varying BMPi conditions (50 ng/ml, 25 ng/ml, 12.5 ng/ml and 6.25 ng/ml). Dashed lines outline the geometrically confined colonies. (D,E) pSMAD1 signaling distribution represented as a function of colony radius (D) and averages of 143,119, 129, 163 colonies for respective conditions (E). Data were pooled from two experiments. (F) Predicted distribution of free BMP4 ligands, following induction with 50 ng/ml BMP4, as a function of colony diameter (700 µm, 600 µm, 500 µm, 400 µm, 300 µm and 200 µm). (G) Graphical depiction of predicted distributions of the data shown in F. (H) Representative immunofluorescence images of colonies stained for pSMAD1 24 h after induction with 50 ng/ml of BMP4. (I) Average pSMAD1 expression of 87, 118, 178, 261, 437 and 373 colonies for respective conditions. Standard deviations are shown in gray, and 95% confidence intervals in black. Scale bars: 200 µm.

To simulate RD-mediated self-organization of pSMAD1 activity, we developed a finite element model that predicts the spatiotemporal distribution of signaling-competent, free BMP4 ligands within the differentiating geometrically confined colonies using the RD-specific two-component, coupled, partial differentiation equation set (Turing, 1952; Gierer and Meinhardt, 1972) (supplementary Materials and Methods, Fig. S15, Fig. S16, Table S3, Movie 1). To determine whether this model could accurately predict experimental data, we performed sweeps on two key model parameters: the initial concentration of BMP4 in the induction medium (BMPi) and the colony size. Our model predicted that reducing BMPi while maintaining the colony diameter at 1000 µm would still lead to the formation of distribution gradients of free BMP4 within the colonies, but with lower ligand levels at the colony periphery (Fig. 4A,B). Consistent with model predictions, the spatial distribution of the pSMAD1 concentration in 1000 µm diameter colonies 24 h after induction with varying BMPi (6.25 ng/ml, 12.5 ng/ml, 25 ng/ml and 50 ng/ml) formed gradients, with lower BMPi conditions producing lower levels of peak pSMAD1 activity at colony peripheries (Fig. 4C-E, Fig. S8). We next queried the model to predict how reducing colony size, at a fixed BMPi dose, would affect the spatial distribution of free BMP4 ligands in the differentiating colonies. The model predicted that reducing the colony size would progressively increase the presence of free BMP4 ligands at the colony centers (Fig. 4F,G). To test the model predictions experimentally, we differentiated colonies of varying sizes with constant BMPi dose (50 ng/ml), and assessed pSMAD1 activity 24 h post-induction. Consistent with the model predictions, we found that small colonies were unable to form regions with low pSMAD1 activity (Fig. 4H,I). These findings are consistent with our hypothesis that the self-organization of pSMAD1 activity observed in differentiating hPSC colonies is governed by a BMP4-NOG RD network.

### Peri-gastrulation-like fates arise in a manner consistent with the PI paradigm

**Fig. 5.**
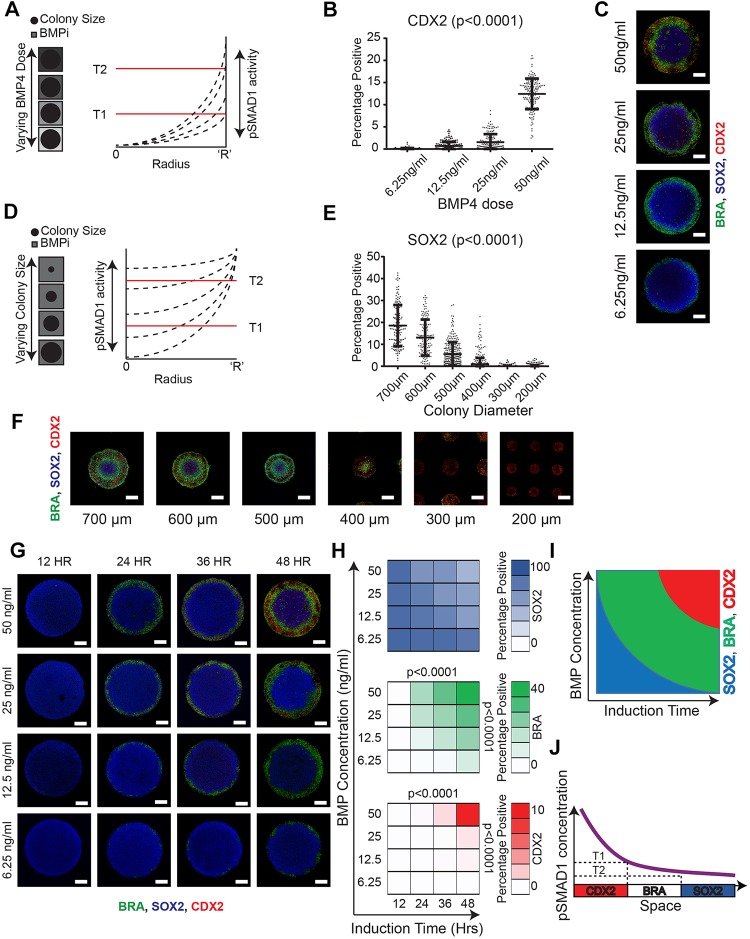
**Fate patterning in hPSC colonies arises in a pSMAD1 threshold**-**dependent manner.** (A) Overview of the experimental setup: varying BMPi while maintaining colony size constant perturbs pSMAD1 signaling at the colony periphery. T1 and T2 represent putative thresholds that determine fate patterning. (B) Quantification of cells expressing CDX2 in 1000 µm colonies induced to differentiate at varying BMPi concentrations (6.25 ng/ml, 12.5 ng/ml, 25 ng/ml and 50 ng/ml). *P*-value calculated using the Kruskal–Wallis test. Number of colonies are 136, 168, 169 and 156 for the respective conditions. Results pooled from two separate experiments. Data are mean±s.d. and individual data points indicate identified colonies. (C) Representative immunofluorescence images of SOX2, BRA and CDX2 expression in geometrically confined 1000 µm diameter colonies differentiated in 6.25 ng/ml, 12.5 ng/ml, 25 ng/ml and 50 ng/ml of BMP4. (D) Overview of the experimental setup: varying colony size while maintaining constant BMPi perturbs the level of pSMAD1 signaling in the colony center. T1 and T2 represent putative thresholds that determine fate patterning. (E) Quantification of cells expressing SOX2 in colonies of varying diameters (700 µm, 600 µm, 500 µm, 400 µm, 300 µm, 200 µm) differentiated in 50 ng/ml BMPi. *P*-value calculated using the Kruskal–Wallis test. Numbers of colonies analyzed from two separate experiments were 144, 160, 279, 466, 789 and 1607 for the respective conditions. Data are mean±s.d. and individual data points indicate identified colonies. (F) Representative immunofluorescence images of SOX2, BRA and CDX2 expression in geometrically confined colonies of varying diameters (700 µm, 600 µm, 500 µm, 400 µm, 300 µm and 200 µm). (G) Representative immunofluorescence images of SOX2-, BRA- and CDX2-stained 1000 µm diameter colonies differentiated at varying BMPi concentrations (50 ng/ml, 25 ng/ml, 12.5 ng/ml and 6.25 ng/ml) and induction times (12, 24, 36 and 48 h). (H) Average percentage of cells expressing SOX2, BRA and CDX2 in hPSC colonies. Each condition had over 140 colonies. Data were pooled from two experiments. *P*-values were calculated for the concentration of 50 ng/ml and induction time of 48 h using the Kruskal–Wallis test. (I) Overview of peri-gastrulation-like fate acquisition arising as a function of both morphogen concentration and induction time. (J) Representation of fate acquisition arising in a manner consistent with the cardinal positional information paradigm. Scale bars: 200 µm.

Notably, the perturbations we tested above resulted in a change in pSMAD1 activity both at the periphery (Fig. 4D) and at the center (Fig. 4I) of the colonies. We reasoned that if fate acquisition in these colonies were a function of pSMAD1 activity thresholds, we would observe fate switches under different conditions. Accordingly, we tested the same conditions above – this time after 48 h – and stained for the fate-associated markers SOX2, BRA and CDX2. First, to perturb the pSMAD1 activity at the colony periphery we varied BMPi doses while keeping the colony size constant (Fig. 5A). Consistent with a threshold-dependent fate acquisition model, we found a significant reduction of CDX2 expression in colonies induced at low BMPi conditions (Fig. 5B,C). Furthermore, when we perturbed the pSMAD1 activity at the colony center by varying colony size at a constant BMPi dose (Fig. 5D), we found that SOX2 expression disappeared in smaller colonies (≤300 µm diameter) (Fig. 5E,F). These data indicate that BMP signaling thresholds dictate fate acquisition in the differentiating geometrically confined hPSC colonies.

The current understanding of PI, however, suggests that transcription factors associated with patterned fates arise not just as a function of the morphogen concentration, but also as a function of induction time, i.e. the fates associated with higher levels of morphogen concentration are induced after longer induction times (Briscoe and Small, 2015; Green and Sharpe, 2015; Dessaud et al., 2008, 2007). Therefore, we set out to investigate if fates in the differentiating hPSC colonies arose as a function of both the level of pSMAD1 activity and the time of induction. Accordingly, we tested four different BMPi doses (50 ng/ml, 25 ng/ml, 12.5 ng/ml and 6.25 ng/ml) and analyzed the patterned fates that emerged at four different induction times (12 h, 24 h, 36 h and 48 h). We found that the BRA and CDX2 fates did not arise consistently at either lower BMPi doses or at shorter durations of higher BMPi doses (Fig. 5G,H, Fig. S9). This analysis suggests that the cell fate patterning in the hPSC colonies mediated by the pSMAD1 gradient follows PI, as reflected by the characteristic PI-like profile (Fig. 5I). In summary, our findings demonstrate that the fate patterning within the differentiating hPSC colonies occurs via the cardinal PI model (Fig. 5J).

### A two-step process of RD and PI governs peri-gastrulation-like fate patterning

**Fig. 6.**
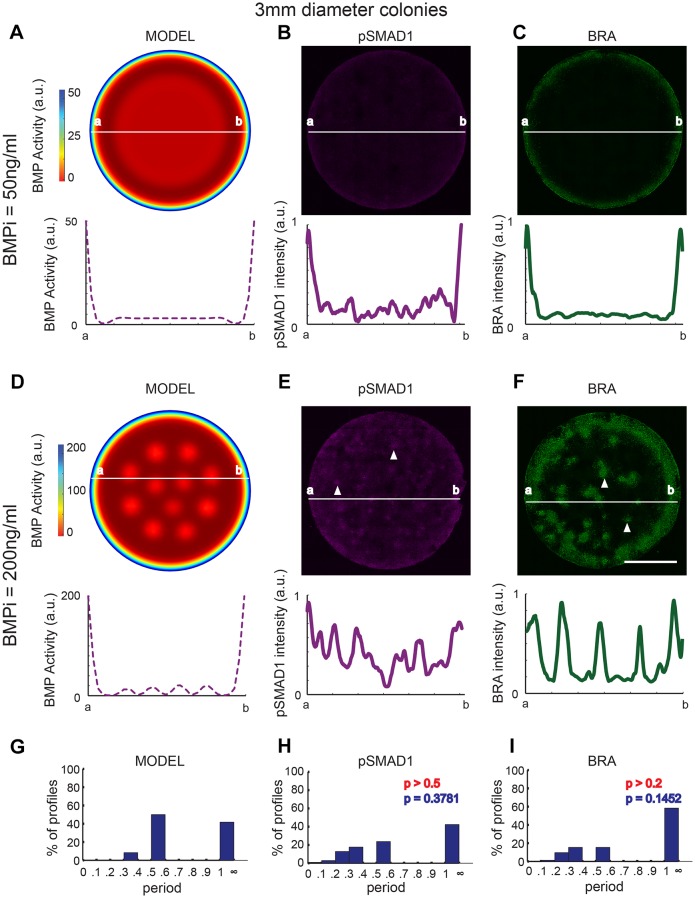
**High BMP4 dose in induction media recapitulates stereotypic RD-like periodic patterns in 3 mm diameter hPSC colonies.** (A) Model predictions for spatial profile of BMP activity for colonies of 3 mm diameter in the presence of 50 ng/ml BMPi. (B,C) Experimental data showing representative immunofluorescence images and spatial expression profiles for pSMAD1 (B) and BRA (C). (D) Model predictions for spatial profile of BMP activity for 3 mm diameter colonies differentiated at a higher dose of BMPi (200 ng/ml). (E,F) Experimental data showing representative immunofluorescence images and spatial expression profiles for pSMAD1 (E) and BRA (F). Arrowheads indicate regions of high pSMAD1, and BRA expression. (G-I) Histogram of dominant periods identified in the computational model of RD-like BMP pattern formation (G), and experimentally identified periods for pSMAD1 (*n*=28 colonies pooled from 3 experiments) (H) and BRA (*n*=47 colonies pooled from 3 experiments) (I) expression. *P*-values in red were calculated using Mann–Whitney *U*-test, and in blue were calculated using Kolmogorov–Smirnov test. Periods greater than 1 indicate no periodicity. Scale bars: 1 mm.

Our data indicate that (1) the formation of the pSMAD1 gradient follows an RD mechanism, and (2) the subsequent fate acquisition follows a PI mechanism, suggesting a two-step process of biological fate patterning in geometrically confined hPSC colonies. Recognizing that the classic RD models have periodic peaks of signaling activity, and patterned fates (Green and Sharpe, 2015; Turing, 1952; Kondo and Miura, 2010), we queried our mathematical model to identify conditions that would result in a periodic distribution of free BMP ligands. Our model predicted that an increase in the colony size alone would be insufficient to induce this periodic distribution (Fig. 6A, Movie 2). Consistent with this prediction, we did not detect a noticeable periodic response in either pSMAD1 levels at 24 h post differentiation with BMP4 (Fig. 6B, Fig. S10A), or BRA expression after 48 h of BMP4 treatment with 50 ng/ml (Fig. 6C, Fig. S11A) when we increased colony diameter from 1000 μm to 3 mm. However, our model predicted that a concomitant increase in the BMP4 dose along with an increase in the colony size would result in the induction of a periodic response of BMP activity (Fig. 6D, Movie 3). Consistent with this prediction, we found that differentiating hPSC colonies of 3 mm diameter with a BMP4 dose of 200 ng/ml resulted in periodicity in both pSMAD1 at 24 h (Fig. 6E, Fig. S10B), and BRA expression at 48 h post induction (Fig. 6F, Fig. S11B). We quantitatively compared the theoretical RD-like periodicity with the experimentally observed behavior to assess the predictive ability of the model. We measured the profile of the predicted BMP activity radially from the center of the colony every 30°, for the condition in which a 3 mm colony was induced to differentiate in the presence of 200 ng/ml of BMP4, and quantified the dominant periods using a 1D Fourier transform. The same analysis was performed for pSMAD1 and BRA expression patterns (Fig. S12) (Materials and Methods). The means and distributions of the theoretically predicted and experimentally observed periods were not significantly different from each other (Mann–Whitney U: *P*>0.5 and *P*>0.2, and Kolmogorov–Smirnov: *P*=0.3781 and *P*=0.1452 for pSMAD1 and BRA, respectively) (Fig. 6G-I), validating the ability of the RD model to predict the experimental RD-like periodic response.

**Fig. 7.**
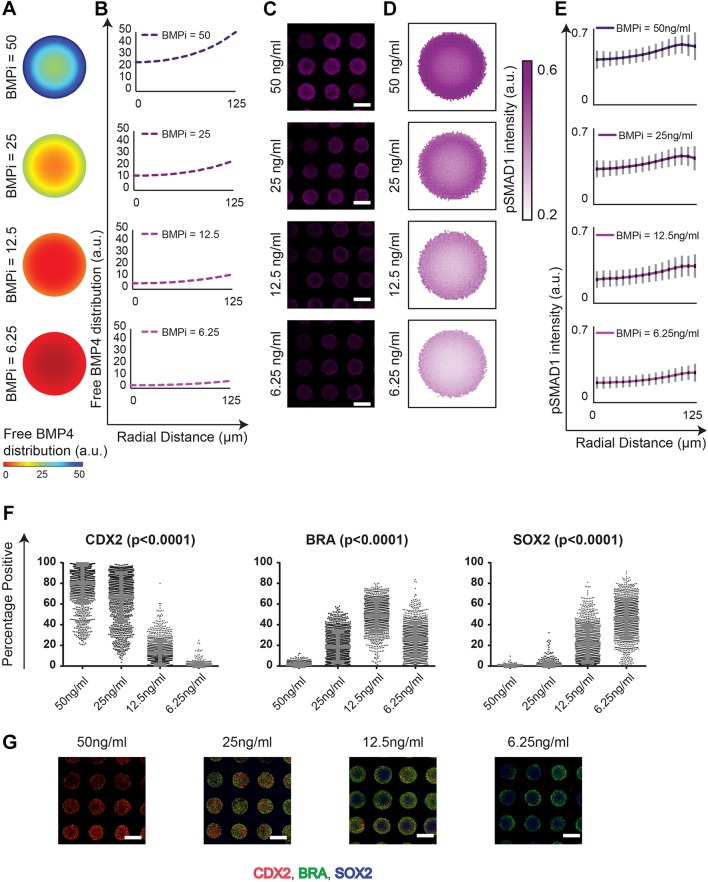
**Low BMP4 dose in induction media rescues fate patterning in 250 µm diameter colonies.** (A) Model predictions for gradient formation of free BMP4 ligands distribution in 250 µm diameter colonies in response to varying BMPi doses. (B) Line plot representation of free BMP4 ligands as a function of the colony radius. (C) Representative immunofluorescence images of colonies stained for pSMAD1 24 h after induction with varying doses of BMP4. (D) Average pSMAD1 expression for 976, 638, 475 and 689 colonies for the respective conditions. (E) Signaling distribution represented as a function of the colony radius. The results were collected from two experiments. Standard deviations are shown in gray, and 95% confidence intervals in black. (F) Quantification of the percentage of cells in each colony expressing CDX2, BRA and SOX2 in 250 µm diameter colonies induced to differentiate at varying BMPi concentrations (6.25 ng/ml, 12.5 ng/ml, 25 ng/ml and 50 ng/ml). *P*-values calculated using the Kruskal–Wallis test. Number of colonies are 1024, 1102 1134 and 1135 for the respective conditions. Results pooled from two separate experiments. Data are mean±s.d. and individual data points indicate identified colonies. (G) Representative immunofluorescence images of SOX2, BRA and CDX2 expression in geometrically confined 250 µm diameter colonies differentiated in 6.25 ng/ml, 12.5 ng/ml, 25 ng/ml and 50 ng/ml of BMP4. Scale bars: 200 µm.

As a final validation, we considered the recently reported claims that ‘edge-sensing’ underlies the observed pattern formation (Etoc et al., 2016). Edge-sensing is a mechanism by which fates are patterned sequentially with the trophectoderm-like fate at the colony edge, followed by the primitive streak-like region, and the ectoderm-like region positioned at the colony center. Consequently, small colonies of 250 µm diameter, which do not have sufficient space from the colony periphery to pattern the primitive streak-like and ectoderm-like regions, would be incapable of fate patterning, only allowing the induction of the trophectoderm-like region (Warmflash et al., 2014). As our interpretation of the data mechanistically implicated a stepwise coordination of RD and PI, our interpretation of the inability of 250 µm diameter colonies to induce all three fates differs from the edge-sensing explanation. Specifically, we argue that at a BMPi dose of 50 ng/ml (the dose tested in the previous reports), RD-mediated organization of free BMP4 ligands within colonies of 250 µm diameter are sustained at high levels throughout the colony (Fig. 4F-I), and past the thresholds that would induce the primitive streak-like and ectoderm-like fates after a 48 h induction (Fig. 5E,F) as per PI. To demonstrate this claim, we queried our model to test whether perturbing BMPi could induce the organization of BMP activity at appropriate levels to rescue the fate patterning of all three lineages. Our model predicted that reducing BMPi would reduce the levels of free BMP4 ligands throughout the colony whereby the BRA- and SOX2-expressing regions might be rescued (Fig. 7A,B). We tested the expression profiles of pSMAD1 activity at 24 h post induction with varying BMPi conditions (50 ng/ml, 25 ng/ml, 12.5 ng/ml and 6.25 ng/ml). Consistent with our model predictions, we observed a reduction of pSMAD1 levels overall, but especially in the center of the colonies (Fig. 7C-E). These data suggested that lower BMPi conditions might rescue the formation of the BRA- and SOX2-expressing regions in colonies of 250 µm diameter in accordance with PI. Indeed, when we tested the fate expression within 250 µm diameter colonies after 48 h of induction with varying BMPi doses, we observed a significant reduction of CDX2 expression at reduced doses of BMP4, and a concomitant increase of BRA and SOX2 expression within the colonies (Fig. 7F,G). Importantly, at a BMPi dose of 12.5 ng/ml, patterning of all three fates is rescued in 250 µm diameter colonies (Fig. 7G).

**Fig. 8.**
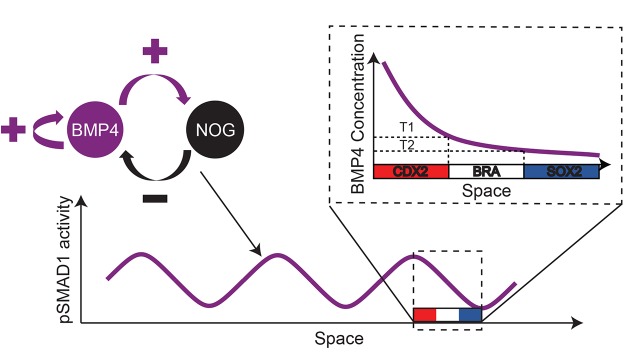
**Mechanism of peri-gastrulation-like fate patterning in geometrically confined hPSC colonies.** A BMP4-NOG RD network induces a radial and periodic pSMAD1 activity gradient in geometrically confined hPSC colonies. The fate acquisition due to the pSMAD1 gradient follows the classical PI paradigm.

Taken together, these data are consistent with our hypothesis that the fate acquisition in the geometrically confined hPSC colonies, in response to BMP4 mediated differentiation, arises via a two-step process of RD and PI (Fig. 8).

## DISCUSSION

### Experimental models to study developmental fate patterning

Of the various proposed models of biological pattern formation, RD and PI have emerged as the prevailing accepted mechanisms to describe tissue organization during early development. RD describes a mechanism by which a symmetric morphogen distribution across a developing tissue self-organizes into a signaling gradient, and PI explains how this gradient is interpreted by the tissue resulting in patterned cell fates. Although both RD (Economou et al., 2012; Sick et al., 2006; Raspopovic et al., 2014) and PI (Gregor et al., 2007, 2008; Houchmandzadeh et al., 2002; Jaeger et al., 2004; Green and Smith, 1990; Chen et al., 2012) have been widely studied independently of each other, few developmental model systems allow for studying both aspects of biological pattern formation. Here, we introduce such a model – a defined *in vitro* platform of developmentally relevant fate patterning using hPSCs to enable studies probing the mechanisms underpinning both RD and PI concurrently.

### Relevance to recent studies for peri-gastrulation-associated fate patterning

Our study closely follows two recent reports in which BMP4-supplemented CM induced similar fate patterning in geometrically confined hPSC colonies as that observed in our work (Warmflash et al., 2014; Etoc et al., 2016). Although we use defined conditions, our observations were remarkably similar to those reports, underscoring the robustness of their findings. Similar to our claims, Etoc et al. (2016) also proposed that BMP4-induced fate patterning in hPSC colonies occurs due to a self-organized pSMAD1 signaling gradient, and suggested that this self-organization is regulated by coordination of two different mechanisms. First, a negative-feedback control of BMP activity mediated by BMP signaling-induced NOG expression; and, second, a density-dependent reduction in sensitivity to BMP4 ligands over the entire colony except for the periphery as a result of re-localization of BMP receptors from apical to basolateral regions (a mechanism called ‘edge-sensing’), rendering them inaccessible and therefore incapable of inducing the BMP signaling pathway. Our interpretation of both our own data and that reported by Etoc et al., however, differs in a few important aspects outlined below.

Although their model of dual negative feedback on BMP signaling mediated by NOG and receptor inaccessibility (Fig. S13A) predicts the formation of a radial signaling gradient, it does not describe a classical RD system. This is because it lacks positive feedback of the morphogen (Turing, 1952; Kondo and Miura, 2010; Murray, 2008; Gierer and Meinhardt, 1972), and would therefore never give rise to periodic RD-like patterns (simplified model shown in Fig. S13B,C). In contrast, we demonstrate that the presence of BMP4 in our cultures, regardless of the basal medium used, elicits both a positive- and a negative-feedback response mediated by the upregulation of BMP4 and NOG, respectively. BMP4 and NOG form a stereotypical activator-inhibitor pair (Gierer and Meinhardt, 1972), as BMP4 can induce short-range activation of BMP signaling (Zhang and Li, 2005), and NOG is a highly diffusible molecule that can result in long-range inhibition of BMP signaling (Smith and Harland, 1992; Inomata et al., 2013). Importantly, we explicitly demonstrate the induction of an RD-like response in larger colonies upon high dose induction of BMP4, both in pSMAD1 activity at 24 h after induction and in the fate acquisition as shown by BRA expression 48 h after induction (Fig. 6, Figs S10 and S11).

Etoc et al. demonstrate that as cell density increases, the BMP receptors re-localize to basolateral regions of the hPSCs. They also demonstrate that these lateralized receptors are incapable of inducing the BMP signaling pathway in hPSCs when BMP4 ligands are presented on the apical side (Etoc et al., 2016). They claim that this mechanism is one of the drivers of the self-organized formation of the pSMAD1 gradient, given that the cell density of the entire colony increases with time. However, they only investigated this at a 500 µm colony diameter. Here, we explored a range of colony sizes, and in 1000 µm diameter colonies we generally observed regions of varying cell densities arising 48 h after BMP4 induction (Fig. 1SE,F, Fig. 2C,D). This finding is somewhat inconsistent with the mechanism proposed by Etoc et al. that the self-organization of a pSMAD1 signaling gradient is induced by density-dependent changes that block BMP signaling. An alternative interpretation to the one put forth by Etoc et al. is that the density-dependent receptor re-localization is in fact a response to the RD-mediated gradient formation, rather than its cause. This interpretation is directly supported by the appearance of RD-like periodic patterns in both pSMAD1 and BRA expression (Fig. 6, Fig. S10) in colonies of 3 mm diameter in which many sporadic spots of higher density relative to the adjacent regions can be observed. In this case, differentiating hPSCs either migrate down a pSMAD1 gradient, or proliferate at different rates depending on the pSMAD1 activity they are exposed to, resulting in regions of varying cell densities. Further work is required to determine which of these possibilities is dominant.

Although our interpretations differ on how ‘edge-sensing’-mediated receptor availability relates to pSMAD1 gradient formation, the ‘edge-sensing’ model proposed by Etoc et al. (2016) nicely parallels our assumed boundary condition for the RD model. For our mathematical RD model, we assumed that the distribution of free BMP4 ligands across the colony evolves over time and space with a fixed concentration of BMP4 at the colony periphery that is equal to the concentration in the bulk medium, a boundary condition that changes depending on the BMP4 concentration of the induction medium. In a recent study, Warmflash et al., claim that the fates are patterned inward from the edge of the geometrically confined colony (Warmflash et al., 2014). Consequently, they contend that 250 μm diameter smaller colonies are incapable of inducing the fate patterning observed in 1000 μm diameter colonies, as the smaller colonies do not have enough space to pattern the primitive streak-like and ectoderm-like regions. However, our interpretation of the ‘edge-sensing’ model identifies conditions that rescue fate patterning of all three fates in 250 μm diameter colonies. Specifically, we demonstrate that in conditions with lower doses of BMP4 in the induction medium, smaller colonies maintain regions that express primitive streak-like and ectoderm-like fates after 48 h of peri-gastrulation-like induction.

### Positional information

We specifically tested and demonstrated that, consistent with the PI paradigm, in the differentiating hPSC colonies fate acquisition arises as a function of both pSMAD1 concentration and induction duration. Notably, although the fate acquisition mediated by the pSMAD1 gradient follows the PI paradigm, the primitive streak-like region is not specified in the absence of Nodal signaling. This underscores that patterning of developing tissues depends on coordination of multiple signaling pathways, reminiscent of quintessential models of PI-mediated fate patterning such as the gap genes in the *Drosophila* that arise from a collaborative stimulation of multiple signals including Bicoid, Caudal, Hunchback, etc. (Briscoe and Small, 2015). Consequently, we propose that although pSMAD1 activity patterns fates according to PI, it does so in conjunction with other transcription factors, for instance SMAD2, which is the effector of Nodal signaling.

### Scaling of morphogen gradients during development

Morphogen gradients formed in developing embryos are thought to scale with size. For instance, the scaling of the dorsal-ventral axis in developing *Xenopus* is robust to dramatic manipulations. When the *Xenopus* embryo undergoes re-section of the ventral half, it still develops into a smaller but proportionally patterned larva (Ben-Zvi et al., 2011). This characteristic has been attributed to the function of the Spemann's organizer (Inomata et al., 2013; Ben-Zvi et al., 2011), underscoring that the robustness of the induction and the stabilization of the morphogen gradients that pattern a developing embryo is the result of a concerted effort by multiple tissues. Our data show that fate organization in differentiating micro-patterned hPSC colonies across varying sizes is not robust to a specific BMP4 concentration. Nevertheless, modulation of the BMP4 induction dose can recapitulate the appropriate patterning. This highlights that robustness to changes in the size of the developing epiblast is attainable, and emphasizes the importance of regulation from other tissues such as the vertebrate organizer (the primitive node) in conferring robustness to a developing human embryo. The mechanism that regulates peri-gastrulation-like pattern formation in the geometrically confined hPSC colonies is one of multiple layers of complexity that would be present in a developing human embryo.

### Identity of fate compartments in the peri-gastrulation-like platform

It has been widely reported that BMP4 treatment of hPSCs gives rise to a population that expresses CDX2. However, the identity of this population has been a subject of debate for nearly two decades. Some groups identify this population as trophectoderm (Xu et al., 2002; Warmflash et al., 2014; Li et al., 2013), whereas other claim that this population is mesodermal in fate (Bernardo et al., 2011; Mendjan et al., 2014; Loh et al., 2016). Interestingly, inhibiting Nodal signaling in the differentiating geometrically confined colonies results in the abrogation of the primitive streak-like population and a significant reduction in CDX2 expression. This suggests that at least a subset of the CDX2-positive population within the patterned peri-gastrulation-like fates require Nodal signaling, and is mesodermal in fate.

Additionally, SOX17 expression in differentiating hPSCs has been widely used as a bona fide marker of the endoderm fate (Warmflash et al., 2014; Blauwkamp et al., 2012; Tsakiridis et al., 2014; Green et al., 2011). However, recent studies have shown that SOX17 is important in the induction of the primordial germ cell (PGC) fate in development (Irie et al., 2015; Kobayashi et al., 2017). Although in our report, consistent with the current literature, we define the SOX17-expressing population as endoderm, we have not ruled out the possibility that this population could be the precursor that subsequently gives rise to PGCs.

Of note, the boundary between the ectoderm-like and the primitive streak-like regions in our platform appears to have cells that express both SOX2 and BRA. We propose two different possibilities that can result in this observation. First, these cells may reflect the population transitioning from a PSC state to a primitive-streak identity, which would be upregulating BRA and downregulating SOX2. Although SOX2 expression in this population would be low, it might still be detectable by immunofluorescence indicating a presence of a SOX2^+^ BRA^+^ phenotype, which would stabilize in a SOX2^−^ BRA^+^ identity. Alternatively, this population might parallel the presumptive neuromesodermal-progenitor-like (NMP-like) population that resides in the node-streak border, caudal lateral epiblast, and the chordoneural hinge sections in the posterior end of the elongating embryo (Gouti et al., 2014; Turner et al., 2014). However, NMPs have been proposed to be present relatively later during gastrulation (Henrique et al., 2015), and whether the SOX2-BRA double-position population in our peri-gastrulation-like model is, in fact, NMP-like requires further investigation.

The fully defined platform we describe here facilitates investigation of the true identity of all these populations in a developmentally appropriate *in vitro* model system.

## Conclusions

In conclusion, we report a defined, *in vitro* model of self-organized human peri-gastrulation-like biological fate patterning, and demonstrate the mechanistic underpinning as a stepwise model of RD and PI paradigms. This *in vitro* model can be employed to investigate the identity of cell populations that arise out of differentiating hPSCs, especially the populations for which identity has been a subject of debate, while maintaining an appropriate developmental context. We further report that fate acquisition that occurs in a manner consistent with PI can require multiple signaling pathways working in concert. Finally, our data implicates the coordination of multiple tissues to induce morphogen gradients that can scale and maintain robustness to perturbations that may occur in the developing embryo. Consequently, our work not only provides deep insight into one of the earliest stages of human embryonic development, but also into general mechanisms involved in patterning of biological form.

## MATERIALS AND METHODS

### Human pluripotent stem cell culture

The CA1 hPSCs (generously provided by Andras Nagy, Samuel Lunenfeld Research Institute, Toronto, Canada) were cultured on Geltrex (Life Technologies, diluted 1:50)-coated 6-well tissue culture plates using mTeSR1 medium (StemCell Technologies) as per manufacturer's instructions. The cells were passaged at a ratio of 1:12 using ReleSR (StemCell Technologies) as per manufacturer's instructions. For the first 24 h after passage, the cells were cultured in the ROCK inhibitor (ROCKi) Y-27632 to increase cell viability. The medium was changed every day and passaged every 4 to 5 days or when the cells reached 75-80% confluence.

### Preparation of PEG plates to micro-pattern hPSC colonies

Custom-sized (110 mm×74 mm) Nexterion-D Borosilicate thin glass coverslips (SCHOTT) were activated in a plasma cleaner (Herrick Plasma) for 3 min at 700 mTorr, and incubated with 1 ml of poly-L-lysine-grafted polyethylene glycol [PLL-g-PEG(5KD), SUSOS] at a concentration of 1 mg/ml at 37°C overnight. The glass slides were then rinsed with ddH_2_O and dried. The desired patterns were transferred to the surface of the PEG-coated side of the coverslip by photo-oxidizing selected regions of the substrate using deep UV exposure for 10 min through a Quartz photomask in a UV-Ozone cleaner (Jelight). Bottomless 96-well plates were plasma treated for 3 min at 700 mTorr and the patterned slides were glued to the bottomless plates to produce microtiter plates with patterned cell culture surfaces. Prior to seeding cells onto the plates, the wells were activated with N-(3-dimethylaminopropyl)-N′-ethylcarbodiimide hydrochloride (Sigma) and N-hydroxysuccinimide (Sigma) for 20 min. The plates were thoroughly washed three times with ddH_2_O, and incubated with Geltrex (diluted 1:150) for 4 h at room temperature on an orbital shaker. After incubation, the plate was washed with PBS at least three times to get rid of any passively adsorbed extracellular matrix (ECM) and seeded with cells to develop micro-patterned hPSC colonies.

### Cell seeding and induction of peri-gastrulation-like fate patterning

To seed cells onto ECM-immobilized PEG-UV 96-well plates, a single-cell suspension of the CA1 line was generated by incubation in 1 ml of TryplE (Invitrogen) per well for 3 min at 37°C. The TryplE was blocked using an equal volume of DMEM+20% KnockOut Serum Replacement (SR) (Invitrogen) and the cells were dissociated by pipetting to generate a single-cell suspension. The cells were centrifuged (200 ***g*** for 5 min) and re-suspended at a concentration of 1×10^6^ cells/ml in SR medium supplemented with 20 ng/ml bFGF (R&D) and 10 µM ROCKi Y-27632. SR medium consists of 74% DMEM, 1% Penicillin/Streptomycin, 1% non-essential amino acids, 0.1 mM β-mercaptoethanol, 1% Glutamax, 2% B27 minus retinoic acid, and 20% SR (all Invitrogen). Wells were seeded in the PEG-patterned 96-well plates at a density of 80,000 cells/well and incubated for 2 h at 37°C. After 2 h, the medium was changed to SR without ROCKi. An alternative seeding process that has also been tested and provides good results is described in a recent protocol (Deglincerti et al., 2016). When confluent colonies were observed (12-16 h after seeding), the peri-gastrulation-like induction was initiated in N2B27 medium supplemented with BMP4 (R&D) (BMP4 dose depended on experimental design). The peri-gastrulation-like pattern formation assay was typically performed with 1000 µm diameter colonies 48 h following induction, although additional time points and colonies sizes were tested as described in the Results section for specific experiments. N2B27 medium consists of 93% DMEM, 1% Penicillin/Streptomycin, 1% non-essential amino acids, 0.1 mM β-mercaptoethanol, 1% Glutamax, 1% N2 Supplement, 2% B27-retinoic acid supplement (all Invitrogen) supplemented with 100 ng/ml Nodal (R&D), and 10 ng/ml bFGF (R&D). Other basal media tested were Nutristem (FroggaBio), mTeSR (StemCell Technologies) and Essential-8 (Life Technologies). Small molecules used to perturb signaling pathways were SB-431542 (StemCell Technologies) and LDN-193189 (StemCell Technologies).

### Single-cell data acquisition and analysis of immunofluorescence imaging

The patterned plates were fixed with 3.7% paraformaldehyde for 20 min, rinsed three times with PBS and then permeabilized with 100% methanol for 3 min. After permeabilization, the patterned colonies were blocked using 10% fetal bovine serum (Invitrogen) in PBS overnight at 4°C. Primary antibodies were incubated at 4°C overnight (antibody sources and concentrations are shown in Table S1). The following day, the primary antibodies were removed, and the plates were washed three times with PBS followed by incubation with the secondary antibodies and DAPI nuclear antibody at room temperature for 1 h. Single-cell data were acquired by scanning the plates using the Cellomics Arrayscan VTI platform using the TargetActivation.V4 bioassay algorithm. This algorithm utilizes the expression intensity in the DAPI channel to identify individual nuclei in all fields imaged and acquires the associated intensity of proteins of interest localized within the identified region. The single-cell data were exported into Context Explorer (CE), a custom software developed in-house for image analysis (J.O., Emanuel J. P. Nazareth, M.T., P.Z., unpublished). In CE, cell colonies are identified through the DBSCAN algorithm as implemented in Python's Scikit-learn package. Within a colony, each cell is assigned *x* and *y* coordinates relative to the colony centroid. To create the colony overlay plots, cells from multiple colonies are grouped in hexagonal bins per their relative *x* and *y* coordinates. These positional bins are color-coded to represent the average protein expression level of all cells within a bin. The color map range is normalized to the lowest and highest expressing hexagonal bins. Spatial expression trends within colonies are also visualized as line plots, in which cells are grouped by the Euclidean distance between a cell and the centroid of the colony. For each colony, the average expression value of all cells within a distance bin is computed. The line plots describe the mean expression value, standard deviation and 95% confidence interval (CI) between colonies as indicated in figure legends. The line plots depicting radial trends of proteins of interest in individual colonies were acquired through the sections of the colonies depicted (shown as white rectangles in the figures) in Fiji (ImageJ). The plot profile extracted was then run through a Savitzky–Golay smoothing filter in Matlab and represented as a function of radial distance.

The quantification of RD-like patterns in differentiating, geometrically confined hPSC colonies of 3 mm diameters was performed using SIESTA, an image analysis platform (Fernandez-Gonzalez and Zallen, 2011; Leung and Fernandez-Gonzalez, 2015), and custom scripts written in MATLAB (Mathworks) using the DIPImage toolbox (TU Delft, The Netherlands). To quantify the periodicity of free BMP4 ligands in the computer model, or pSMAD1 and BRA signals in colonies, we used the Fourier transform. To avoid quantifying background noise, we thresholded the BRA and pSMAD1 signals, and the pixels with intensity lower than the mean of the image plus one standard deviation were set to zero. We measured the signal intensity in the colonies along radii separated by an angle of 30°. The signals were detrended by subtracting the mean pixel value, and smoothened using a Gaussian of σ=3 pixels for the colonies and σ=20 for the model, consistent with the different spatial resolution of experimental and model images. The edge of the BRA colonies was excluded from the analysis to avoid artifacts generated by accumulation of migrating cells. Finally, we used the Fourier transform to decompose each signal into its constituent frequencies and computed a characteristic period, defined as the inverse of the dominant frequency. To be able to compare periods between colonies and the model, the length of each radius was normalized to 1. Periods >1 indicated lack of periodicity.

### Quantitative PCR analysis

RNA extraction was performed using Qiagen RNAeasy miniprep columns according to the manufacturer's protocol, and the cDNA was generated using Superscript III reverse transcriptase (Invitrogen) according to the manufacturer's instructions. The generated cDNA was mixed with primers for the genes of interest and SYBR green mix (Roche, Sigma) and the samples were run on an Applied Biosystems QuantStudio 6 flex real-time PCR machine. Relative expression of described genes was determined by the delta–delta cycle threshold (ΔΔCt) method with the expression of *GAPDH* as an internal reference. Primer sequences used are provided in Table S2.

### Statistics and data analysis

All gene expression results were expressed as mean (+s.d.). Statistical tests for gene expression results were performed using two-tailed Student's *t*-test assuming unequal variance between datasets. The statistical significance for fate acquisition results were calculated either using one-way ANOVA (Dunnett's post hoc test) or the Kruskal–Wallis test as described in the legends. The calculations for Student's *t*-tests were performed in Excel. The one-way ANOVA and the Kruskal–Wallis test calculations were performed in Prism. To evaluate sample means of periodic distributions predicted by the RD model with the distributions experimentally observed, we used a non-parametric Mann–Whitney *U*-test; the Kolmogorov–Smirnov test was used to compare sample distributions.

### siRNA transfection protocol

All siRNA transfection was performed with CA1 hPSCs seeded on either PEG plates as described above, or 24-well plates for the qPCR control experiments to validate the siRNA specificity. NOG-specific siRNAs used were NOG Silencer (Thermo Fisher) and siRNA NOG (Santa Cruz Biotechnology), mixed at 1:1 ratio, at a total concentration of 40 nM, or scrambled control siRNA (Santa Cruz Biotechnology) at a concentration of 40 nM. Transfection was performed using EditPro Stem Transfection Reagent (MTI-Globalstem). Specifically, 1 μl (per 24 well) or 0.2 μl (per 96 well) EditPro Stem Transfection Reagent was diluted with 50 μl or 10 μl Opti-MEM I reduced serum medium (Thermo Fisher), respectively. Diluted reagent was incubated with siRNA for 15 min at room temperature and subsequently added to cells containing 500 μl (24 well) and 100 μl (96 well) culture medium without Penicillin/Streptomycin. Medium was replaced with N2B27+BMP4 18 h after transfection. Cells were analyzed 24 h after transfection by qPCR (24 well) for *NOG* gene expression, or by microscopy (96 well) for pSMAD1 spatial trends.

## Supplementary Material

Supplementary information

Supplementary information
