# Supplementary material for: A stepwise model of reaction-diffusion and positional information governs self-organized human peri-gastrulation-like patterning
Source: Development. 2017 Dec 1;144(23):4298–312. doi: 10.1242/dev.149658 (PMC5769627; doi:10.1242/dev.149658)
Supplement: Supplementary information [file develop-144-149658-s1.pdf]

Movies

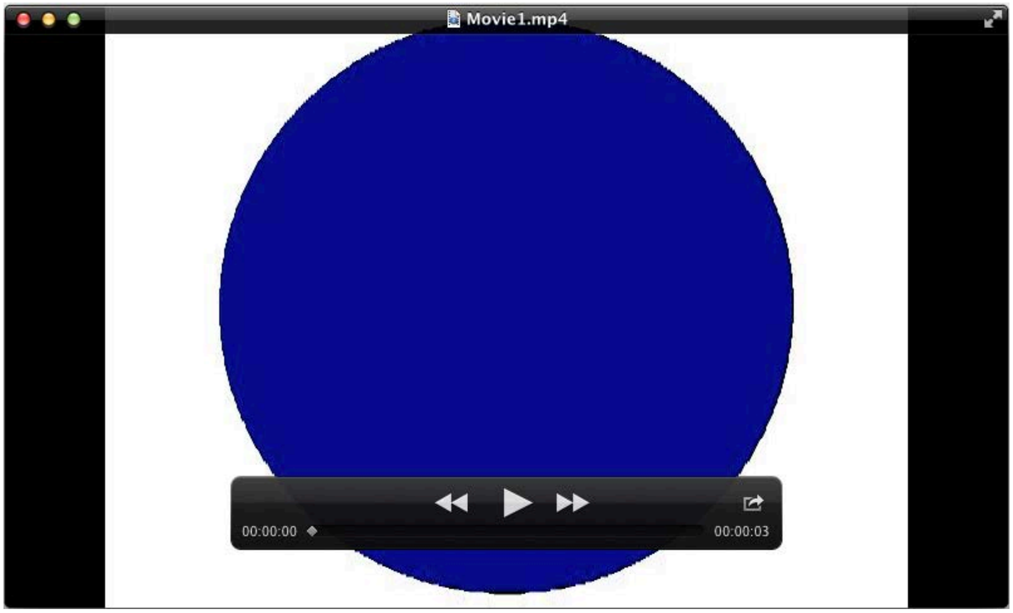

Movie 1: Reaction-Diffusion model of free BMP4 distribution in differentiating hPSC colonies. The video shows the evolution of free BMP4 distribution in differentiating hPSC colonies in accordance with the BMP4-NOGGIN RD model in a differentiating colony of a 1000µm diameter.

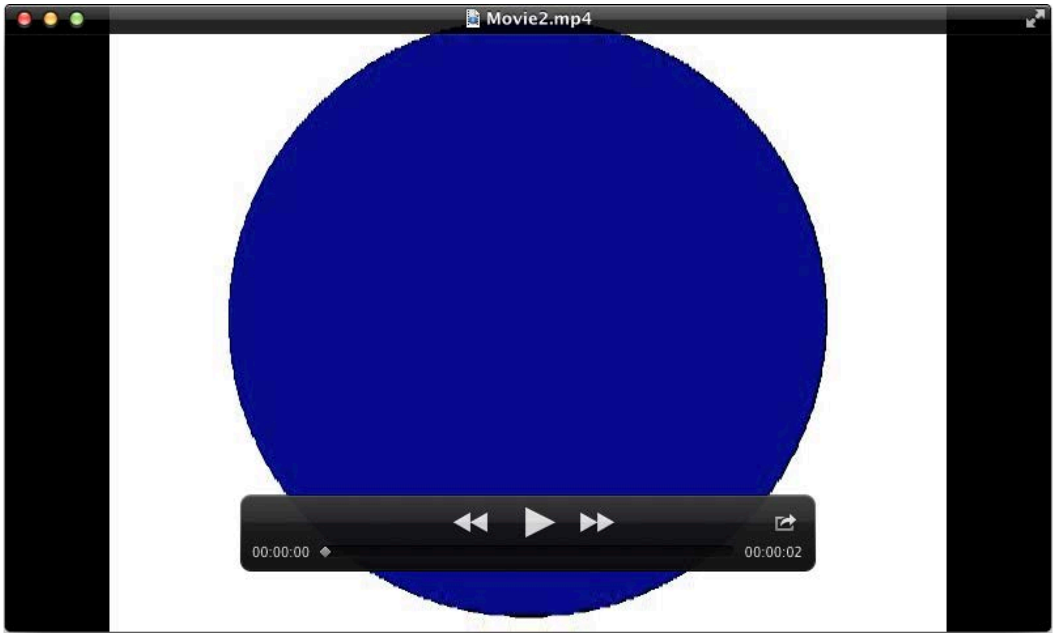

Movie 2: Reaction-Diffusion model of predicts BMPi = 50ng/ml would not generate periodic patterns of free BMP4 distribution in differentiating hPSC colonies of 3mm in diameter. The video shows the evolution of free BMP4 distribution in accordance with the BMP4-NOGGIN reaction diffusion model in differentiating hPSC colonies of 3mm in diameter when induced to differentiate with BMPi = 50ng/ml.

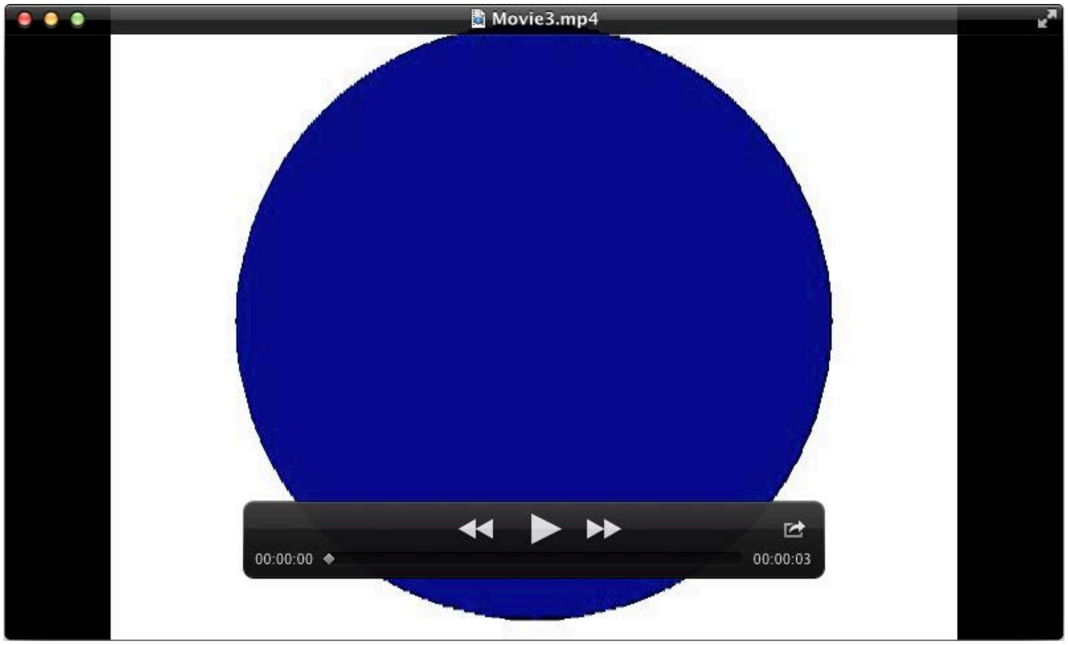

Movie 3: Reaction-Diffusion model of predicts induction of periodic waves of free BMP4 ligands distribution when hPSC colonies of 3mm in diameter are differentiated in BMPi = 200ng/ml. The video shows the evolution of free BMP4 distribution in accordance with the BMP4-NOGGIN reaction diffusion model in differentiating hPSC colonies of 3mm in diameter when induced to differentiate with BMPi = 200ng/ml.

Supplementary Tables

Table S1: Antibodies used for immunofluorescence experiments

Antibody Target	Company and catalog ID	Dilution
CDX2	Abcam (ab15258), Cedarlane (MU392A-UC)	Abcam (1:50), Cedarlane (1:400)
BRA	R&D (AF2085)	1:500
SOX2	Cell Signaling (3579S), R&D (MAB2018)	Cell Signaling (1:200), R&D (1:500)
SOX17	R&D (AF1924)	1:500
EOMES	Abcam (ab23345)	1:500
EPCAM	R&D (SC026) Kit	1:10
SNAIL	R&D (SC026) Kit	1:10
pSMAD1	Cell Signaling (9516S)	1:200
NANOG	Cell Signaling (4903S)	1:200

Table S2: Primers used in qPCR experiments

Primer target	Sequence or catalog ID
BMP4 (fwd)	ATGATTCCTGGTAACCGAATGC
BMP4 (rev)	CCCCGTCTCAGGTATCAAACT
NOGGIN (fwd)	NM_005450.4 (GeneCopoeia Cat # HQP054071)
NOGGIN (rev)	NM_005450.4 (GeneCopoeia Cat # HQP054071)
CHORDIN (fwd)	NM_001304473.1 (GeneCopoeia Cat # HQP067561)
CHORDIN (rev)	NM_001304473.1 (GeneCopoeia Cat # HQP067561)
FOLLISTATIN (fwd)	NM_013409.1 (GeneCopoeia Cat # HQP000565)
FOLLISTATIN (rev)	NM_013409.1 (GeneCopoeia Cat # HQP000565)
GDF3 (fwd)	GTACTTCGCTTTCTCCCAGAC
GDF3 (rev)	GCCAATGTCAACTGTTCCCTT
CERL (fwd)	CTTCTCAGGGGGTCATCTTG
CERL (rev)	TCCCAAAGCAAAGGTTGTTC

Development • Supplementary information

Supplementary Figures

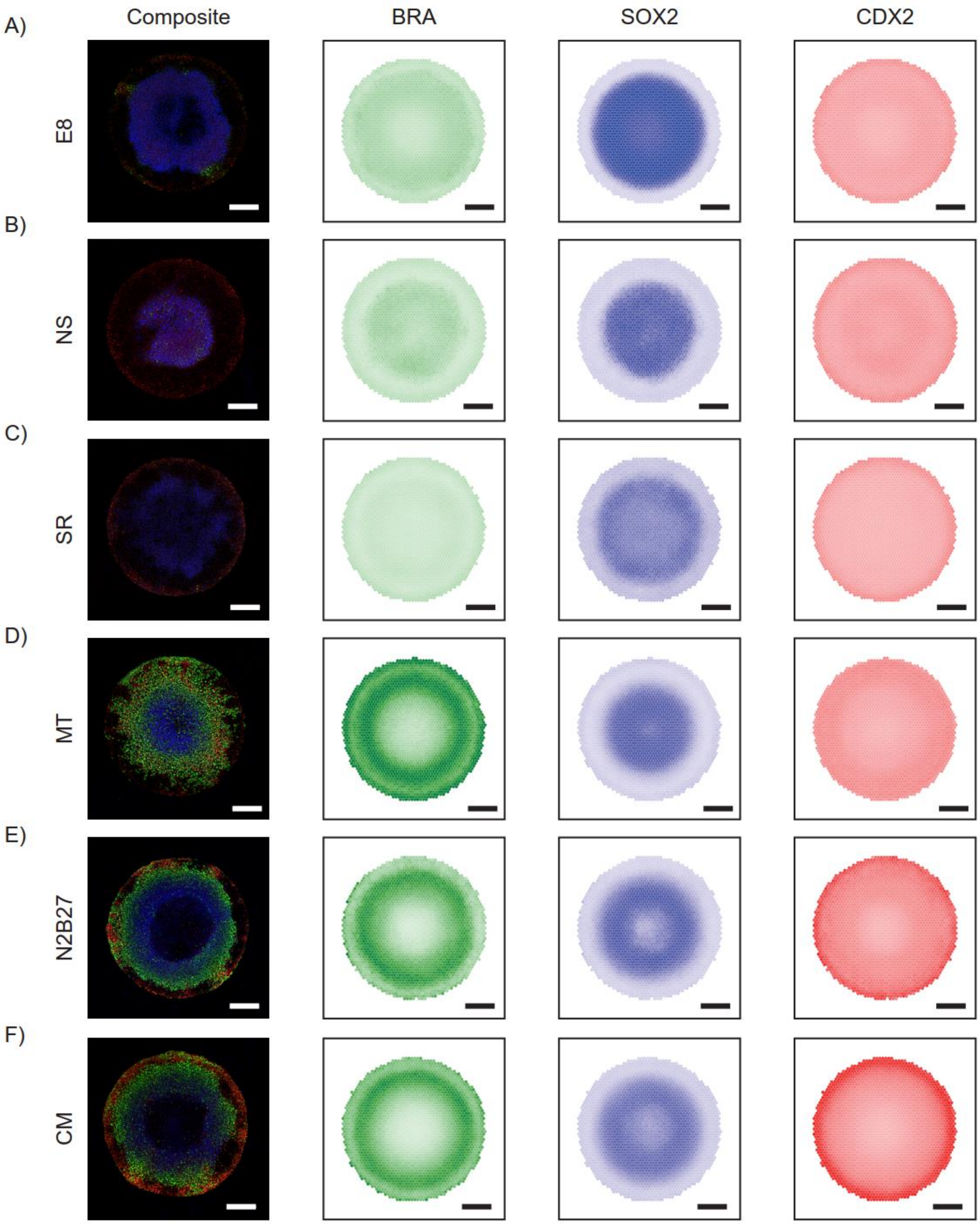

Development • Supplementary information

Figure S1: Peri-gastrulation-like fate patterning in multiple basal medium conditions. Representative composite images and spatial expression average for SOX2, BRA and CDX2 staining in geometrically-confined hPSC colonies differentiated in BMP4 supplemented A) E8, B) Nutristem (NS), C) SR, D) mTeSR, E) N2B27, and F) CM. Scale bars represent 200µm.

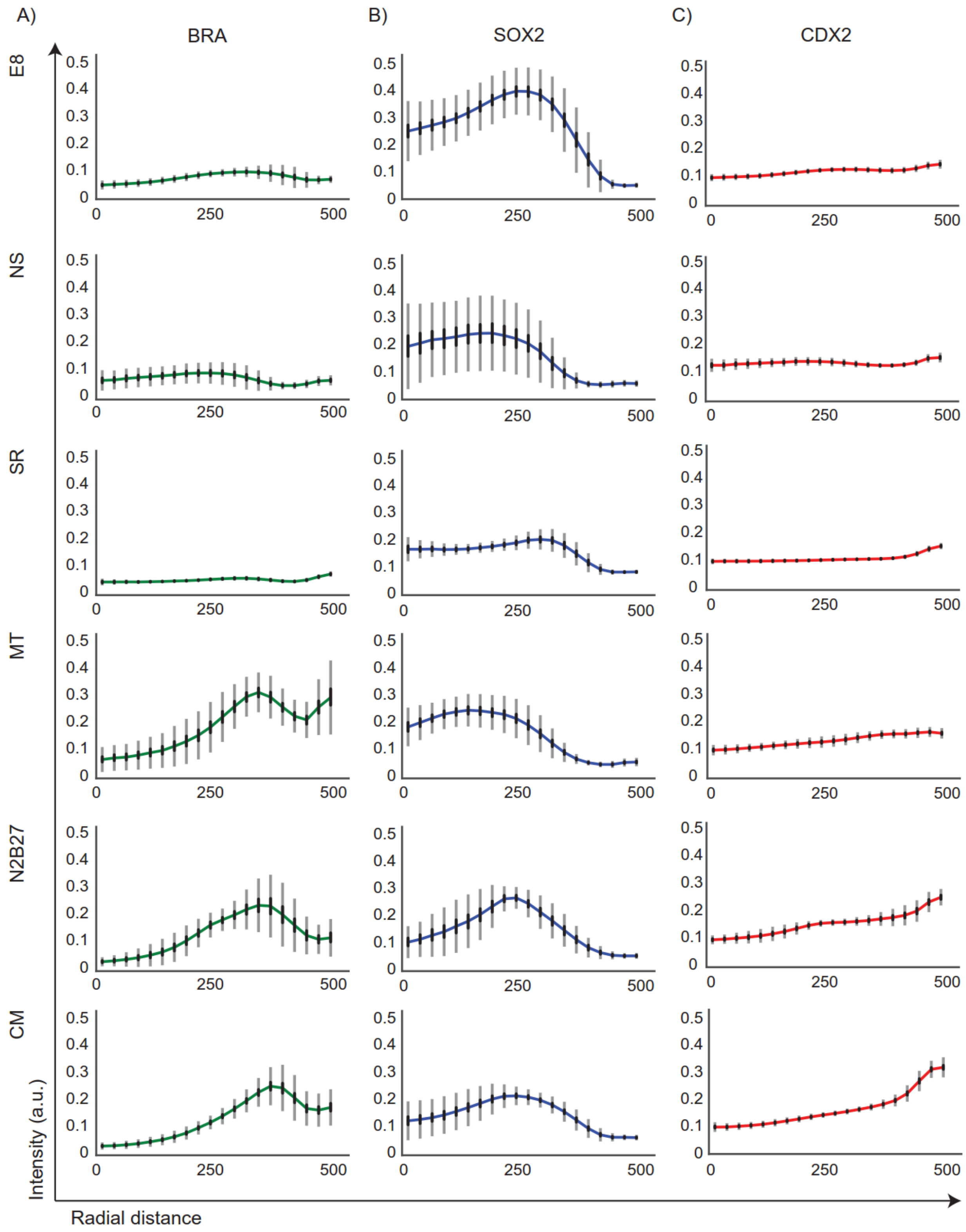

Development • Supplementary information

Figure S2: Spatial trends of CDX2, BRA, and SOX2 observed in different basal media. Radial trends of A) BRA, B) SOX2, and C) CDX2 in BMP4 supplemented E8, NS, SR, MT, N2B27, and CM. Standard deviations shown in grey, and 95% confidence intervals shown in black.

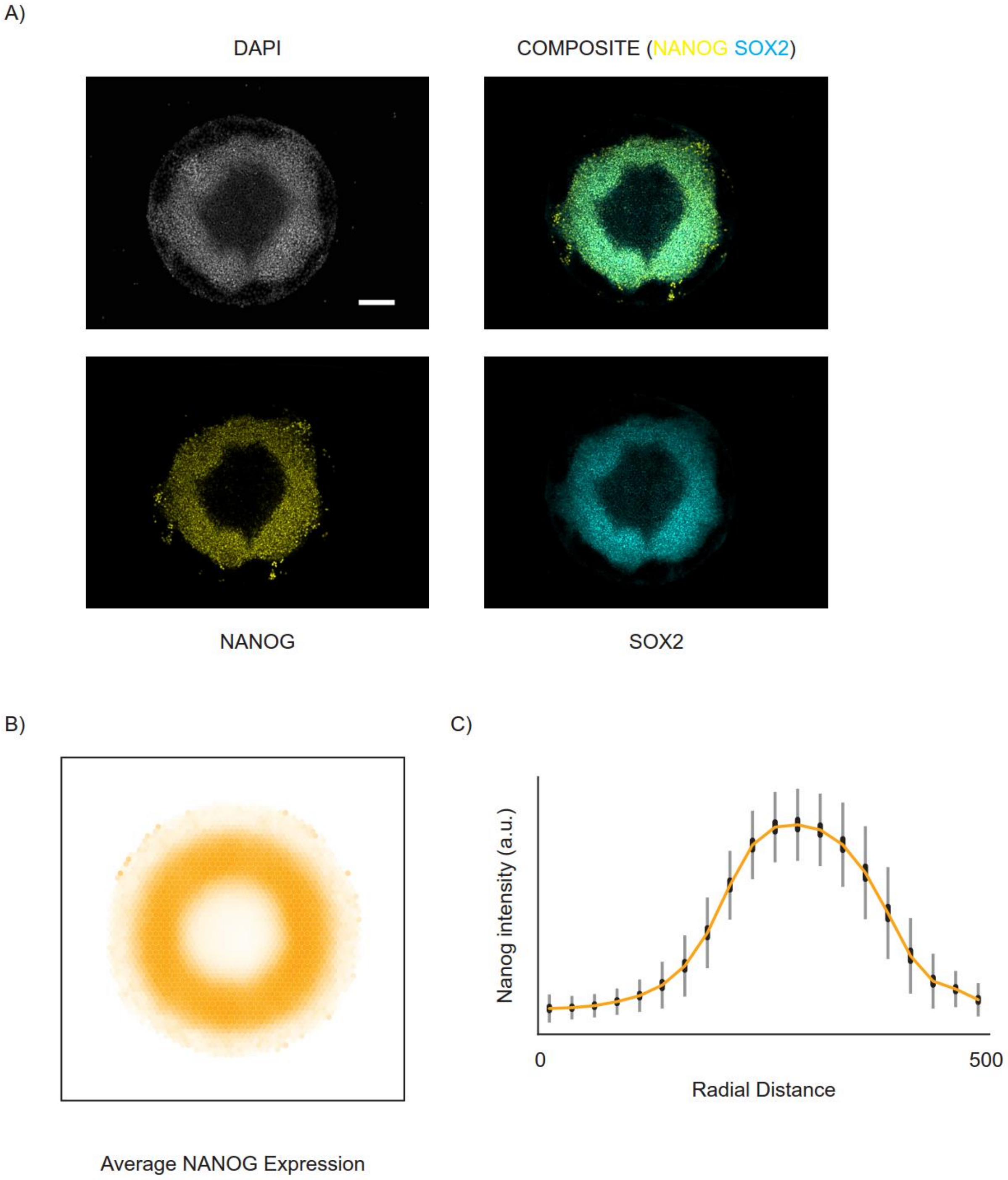

Figure S3: Nanog does not co-localize with SOX2 expression at the center of differentiating hPSC colonies. A) Representative immunofluorescent images of colonies stained for DAPI, SOX2, and NANOG of geometrically confined hPSC colonies cultured in BMP4

Development • Supplementary information

supplemented N2B27. NANOG expression of 95 colonies shown as B) Average map, and C) line-plots of the average radial trend. Standard deviations shown in grey, and 95% confidence intervals shown in black. Data pooled from two experiments. Scale bars represent 200µm.

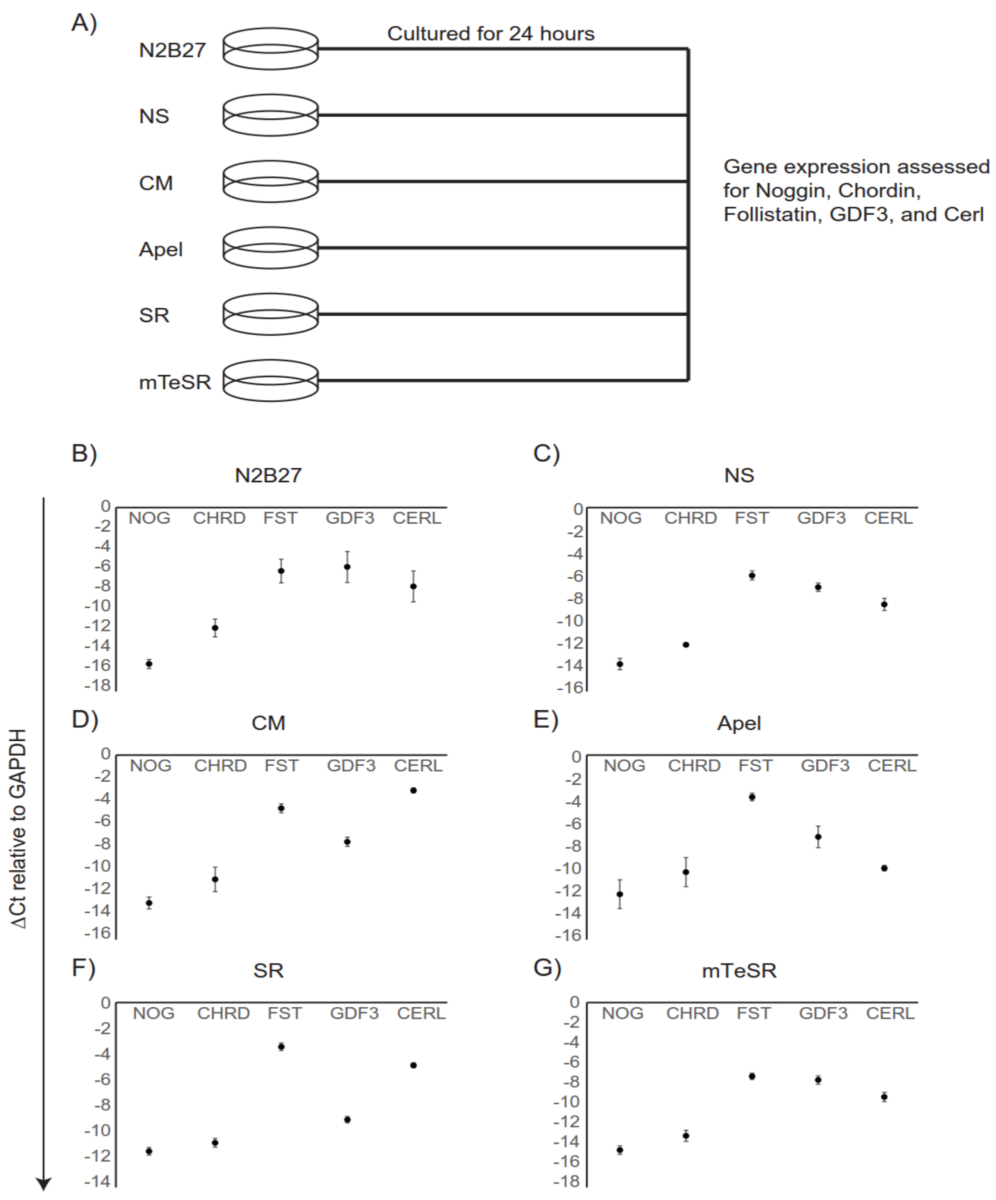

Figure S4: Basal expression of BMP inhibitors in hPSCs during routine culture. A) Overview of experimental setup – hPSCs were cultured in a variety of media conditions for 24 hours and gene expression was assessed for candidate inhibitors of BMP signaling. Expression of various inhibitors (NOG – NOGGIN, CHRD – CHORDIN, FST – FOLLISTATIN, GDF3, and CERL – CERBERUS-Like) of BMP signaling under basal conditions shown as ΔCt relative to GAPDH for CA1 cells cultured in B) N2B27, C) Nutristem (NS), D) Conditioned Medium (CM), E) Apel, F) Serum Replacement medium (SR), and G) mTeSR for 24 hours. Data are shown as mean (S.D) for three independent experiments

Development • Supplementary information

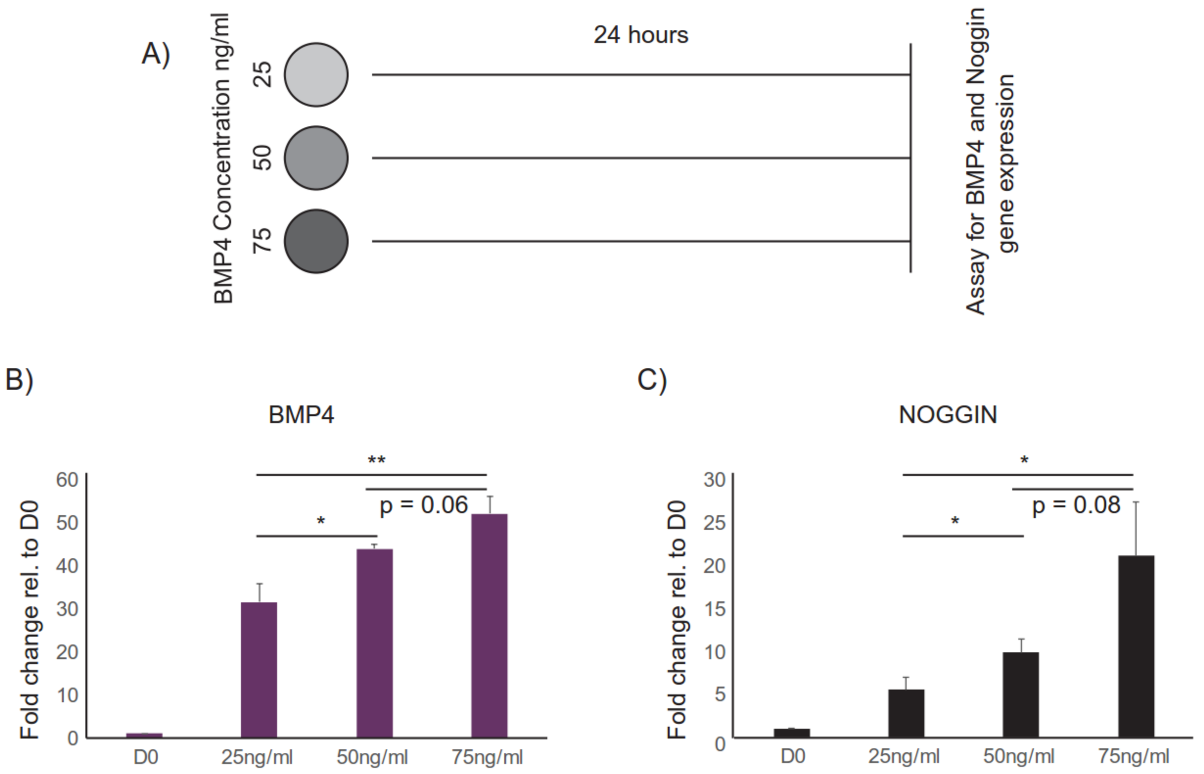

Figure S5: BMP4 and NOGGIN upregulation occur in a BMP4 induction dose-dependent manner. A) Experimental overview: Gene expression data gathered at 24 hours following induction at varying concentrations of BMP4. B) BMP4-induced expression of BMP4. C) BMP4 induced expression of NOGGIN. Data represented as mean and S.D. of three biological replicates. * $p<0.05$, ** $p<0.01$.

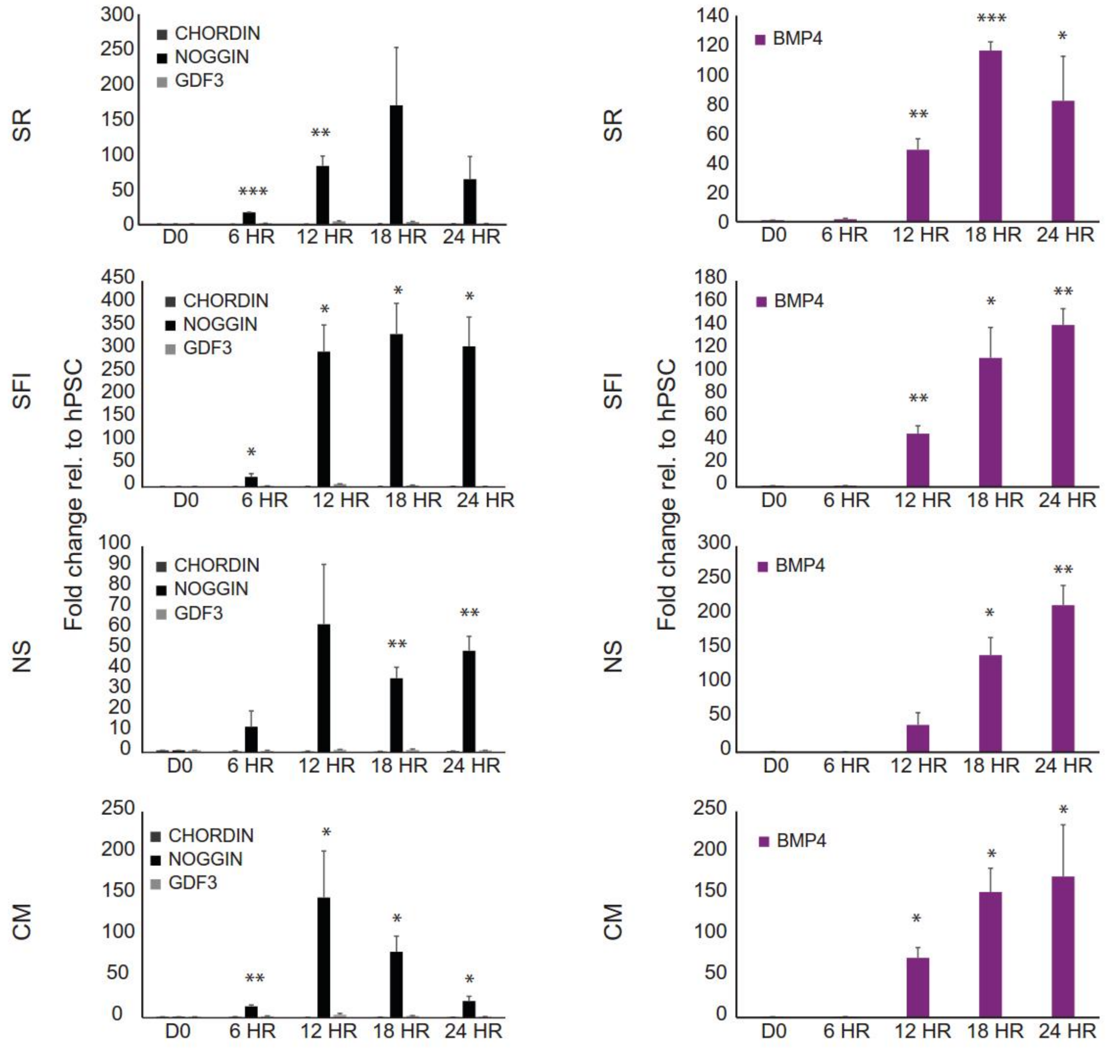

Figure S6: BMP4 induced upregulation of BMP4 and NOGGIN in tested medium conditions. Kinetic gene expression profiles for BMP4 and its cardinal inhibitors in response to BMP4 induced differentiation. Medium conditions tested include a Knockout serum – based medium (SR), a serum-free medium (SFI – for composition, please see Nazareth *et al*., Nature Methods 2013), Nutristem (NS), and Mouse Embryonic Fibroblast conditioned medium (CM). Data represented as mean and S.D of three biological replicates. * $p<0.05$, ** $p<0.01$, *** $p<0.001$.

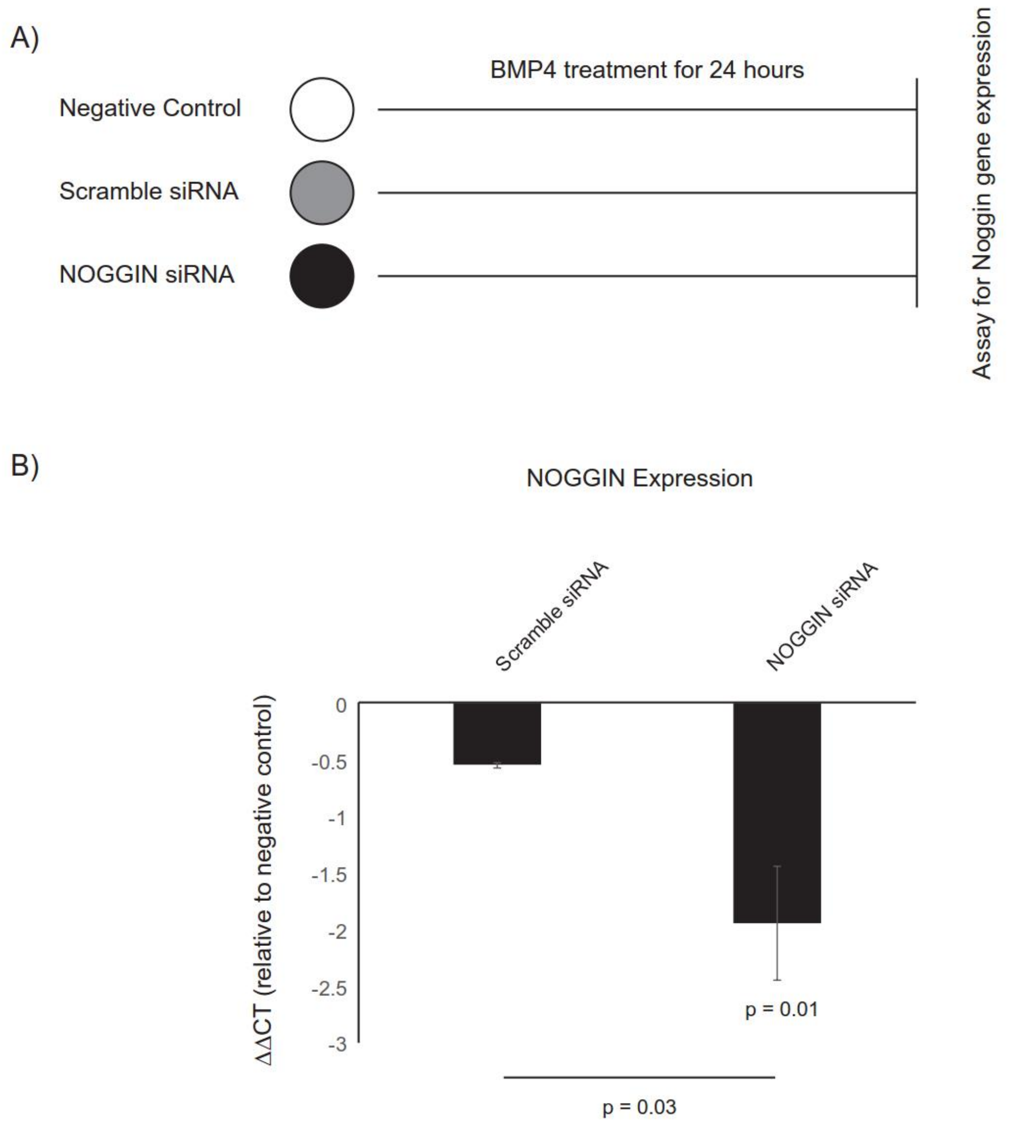

Figure S7: Controls for NOGGIN and Scramble siRNA. A) Overview of experimental setup. hPSC cultures were treated with Scramble, and NOGGIN siRNA with BMP4 for 24h. B) NOGGIN gene expression for the Scramble and NOGGIN siRNA relative to the negative control shown as ΔΔCT. Data represented as mean (s.d.) of three biological replicates. The p values were calculated using Student's t-test.

Development • Supplementary information

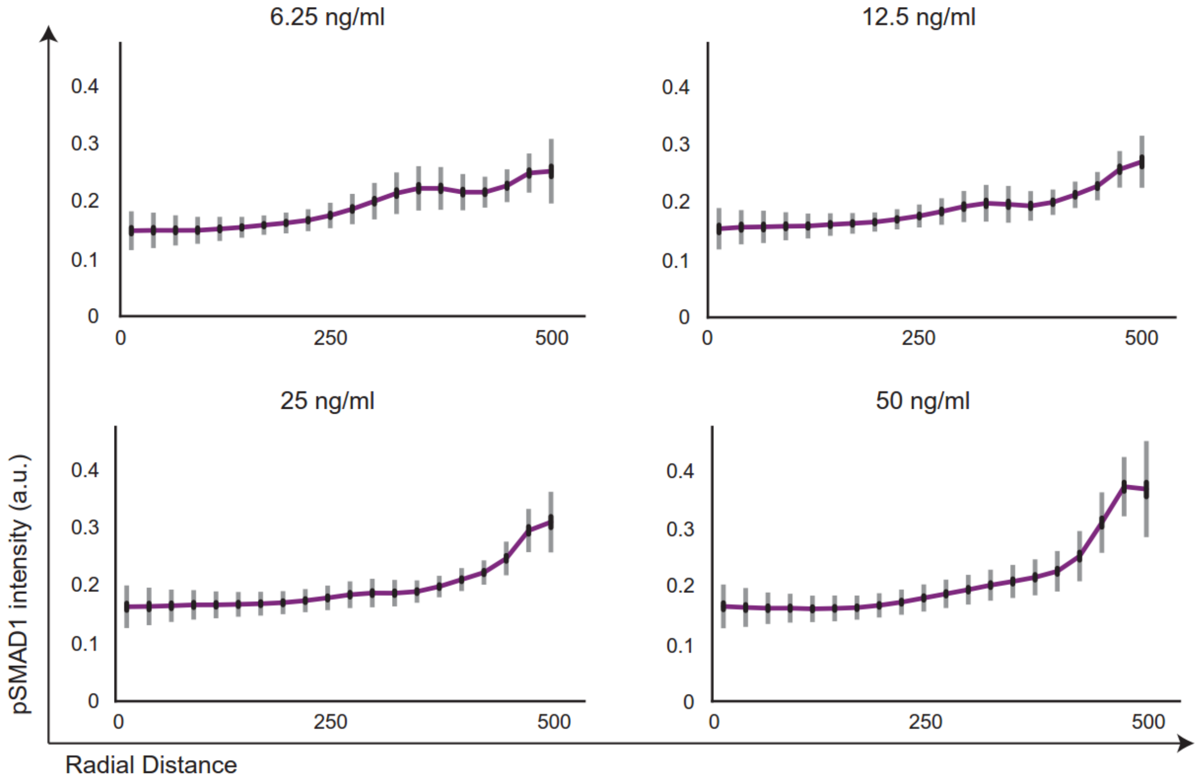

Figure S8: Quantified radial trends of pSMAD1 activity at 24 hours after induction with varying concentrations of BMP4. Radial trends of pSMAD1 activity were observed in varying BMPi concentrations (6.25 ng/ml, 12.5 ng/ml, 25 ng/ml, and 50ng/ml). Standard deviations shown in grey and 95% confidence intervals shown in black.

Development • Supplementary information

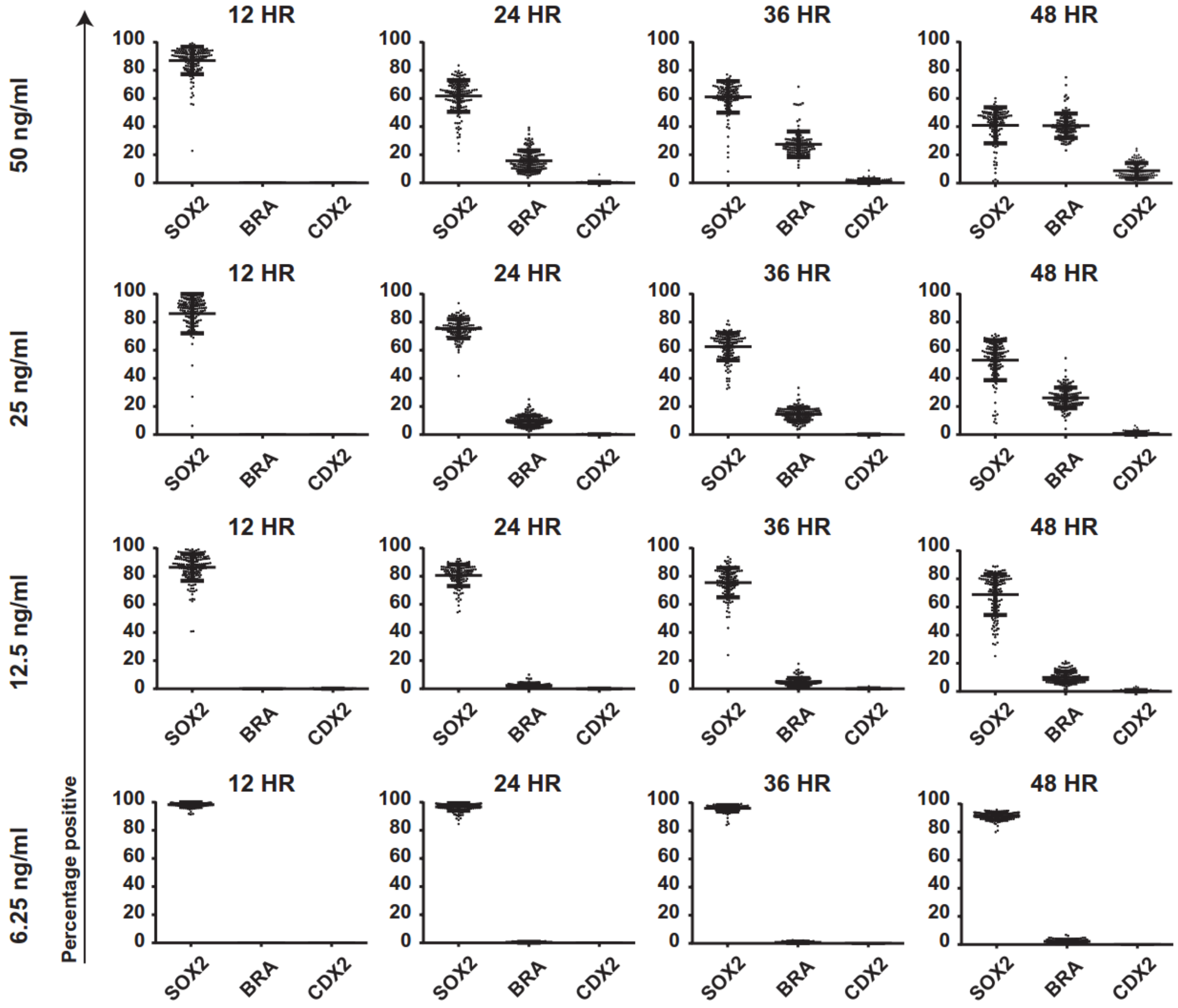

Figure S9: CDX2 and BRA expression in colonies arise as a function of BMP4 dose, and induction time. Percentage of cells expressing SOX2, BRA, and CDX2 in colonies induced to differentiate at varying concentrations of BMP4 (6.25 ng/ml, 12.5 ng/ml, 25 ng/ml, and 50 ng/ml) and induction times (12 hours, 24 hours, 36 hours, and 48 hours). Each condition had over 140 colonies. Data pooled from two experiments.

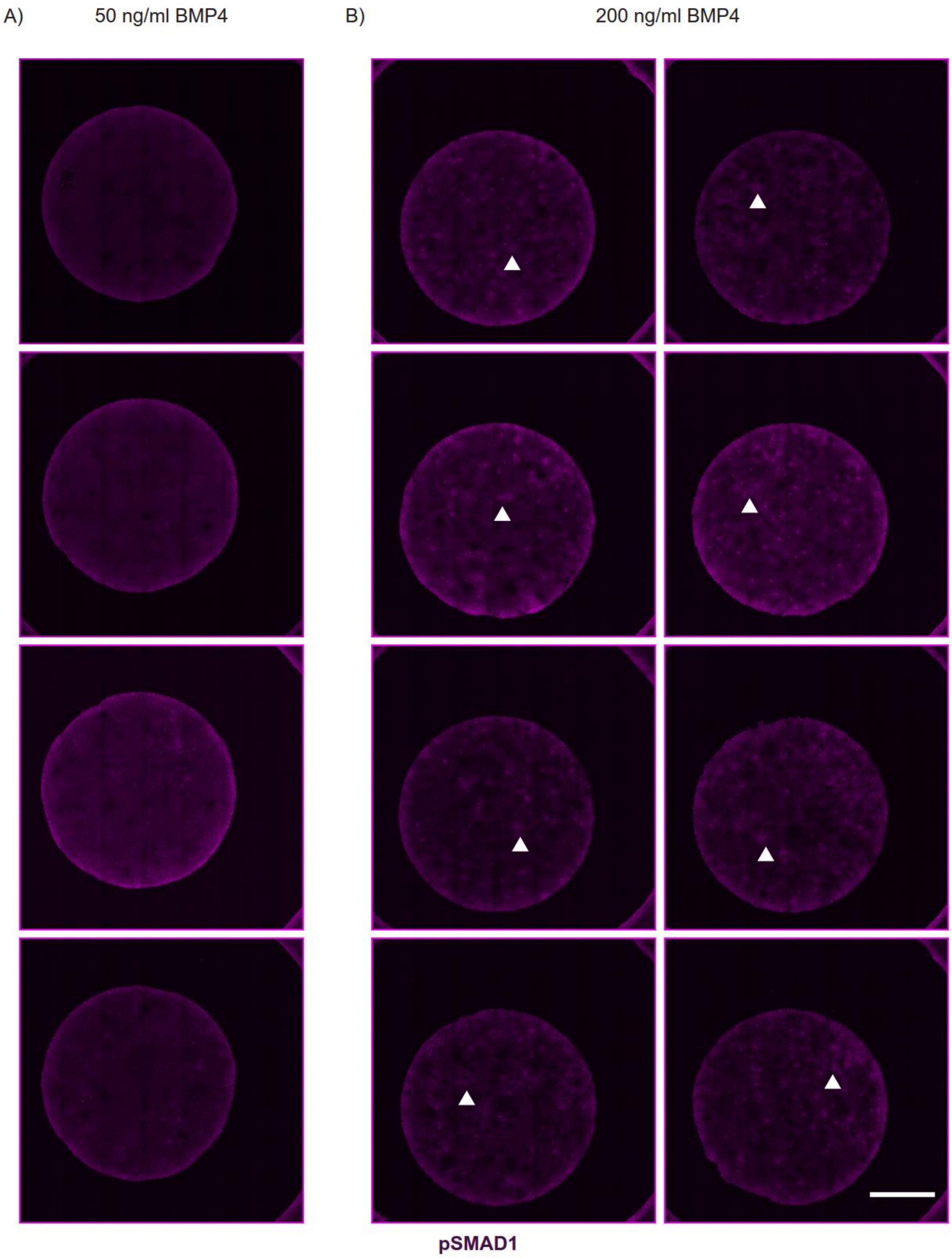

Figure S10: RD-like patterns noted in pSMAD1 activity in 3mm colonies when differentiated with high doses of BMP4. Representative immunofluorescent images of 3mm

Development • Supplementary information

diameter colonies stained for pSMAD1 for a BMP4 dose of A) 50ng/ml, and B) 200ng/ml. White arrowheads denote representative areas of high pSMAD1 activity indicative of RD-like patterns. Scale bars represent 1mm.

Development • Supplementary information

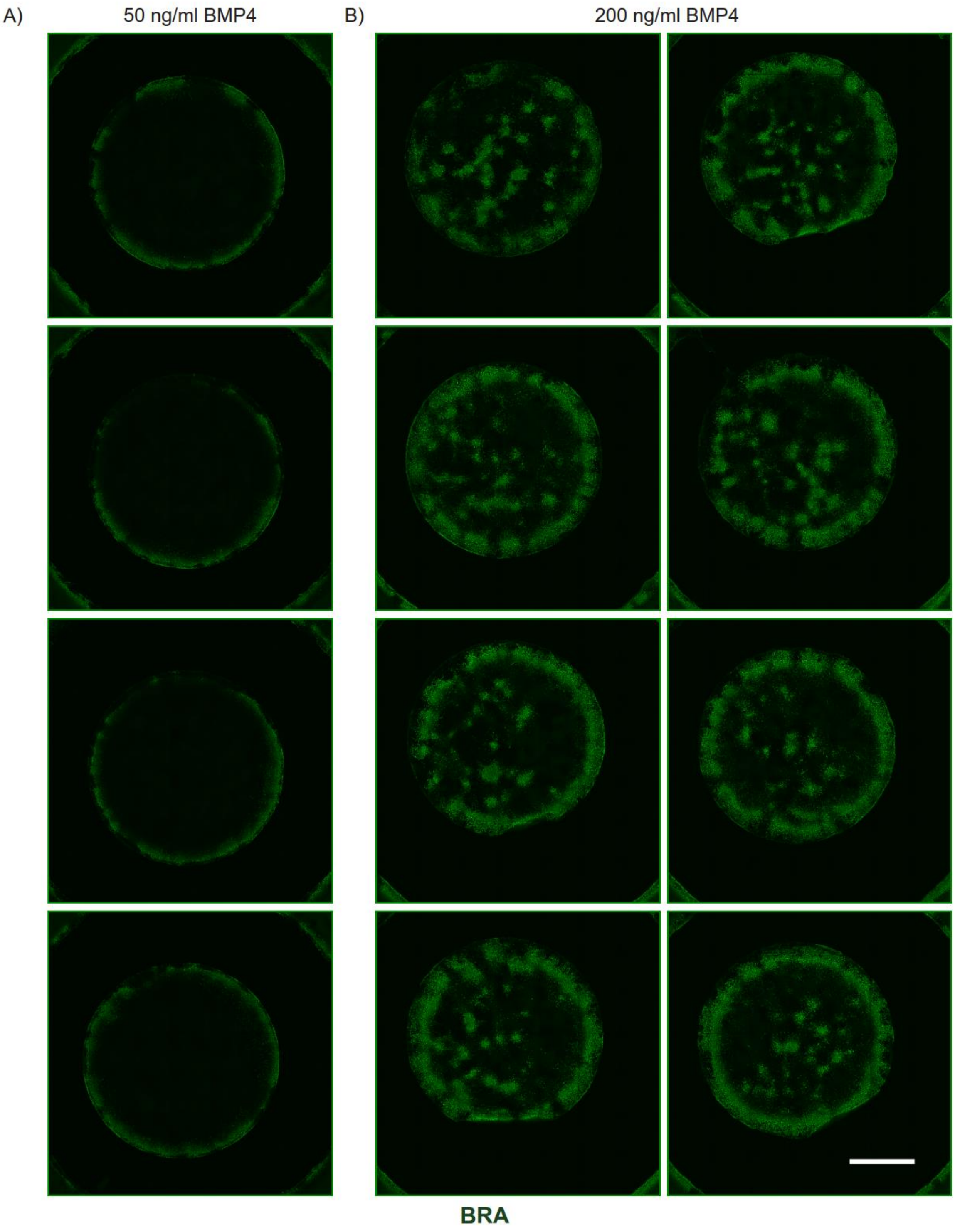

Figure S11: RD-like patterns noted in BRA fate acquisition in 3mm colonies differentiated in high doses of BMP4. Representative immunofluorescent images of 3mm diameter colonies stained for BRA for a BMP4 dose of A) 50ng/ml, and B) 200ng/ml. Scale bar represents 1mm.

Development • Supplementary information

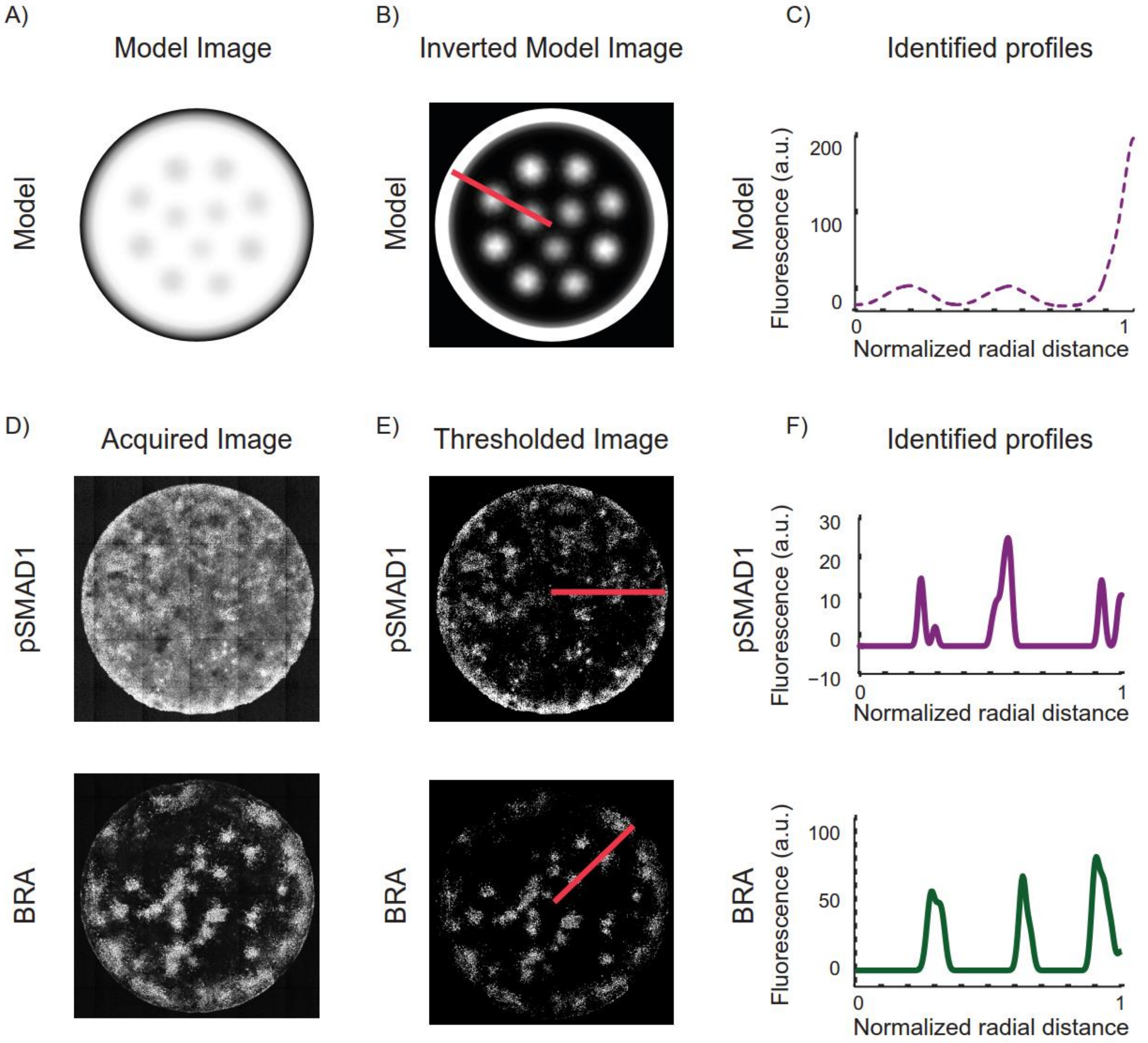

Figure S12: Analysis pipeline for extracting dominant periods of theoretically predicted distribution of free BMP4 ligands and expression patterns in experimental colonies. A) Model prediction, and the inverted image (B) shown in grey scale. Profiles extracted every 30 degrees. C) Profile observed along red line in B. Acquired (D), and thresholded (E) (to remove background noise) images for pSMAD1, and BRA. Profiles extracted every 30 degrees for multiple colonies (n = 28 for pSMAD1, and n=47 for BRA). F) Identified periodic profile along the red lines in (E) for pSMAD1 and BRA.

Development • Supplementary information

A)

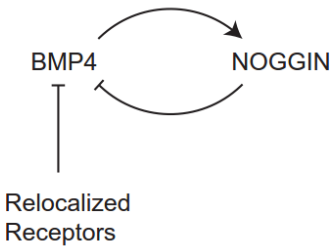

B)

$$\frac{\partial bmp}{\partial t} = -k_1 nog - k_2 - k_3 bmp + D_{BMP}\nabla^2 bmp$$

$$\frac{\partial nog}{\partial t} = k_4 bmp - k_5 nog + D_{NOG}\nabla^2 nog$$

All coefficients (k1, k2, k3, k4, k5 DBMP, and DNOG) are positive.
k_1 – inhibition of BMP signaling mediated by NOGGIN.
k_2 – Inhibition of BMP signaling mediated by relocalized receptors.
k_3 – Degradation rate of BMP4.
k_4 – Production rate of NOGGIN mediated by BMP signaling.
k_5 – Degradation rate of NOGGIN.
D_{BMP}, and D_{NOG} – Diffusivities of BMP4 and NOGGIN.

C)

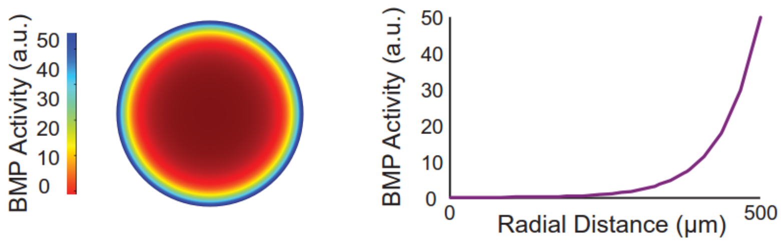

k_1 = 0.01 [1/s]
k_2 = .003 [1/s]
k_3 = .003 [1/s]
k_4 = .008 [1/s]
k_5 = .009 [1/s]
D_{BMP} = 10μm²/s
D_{NOG} = 50μm²/s

D)

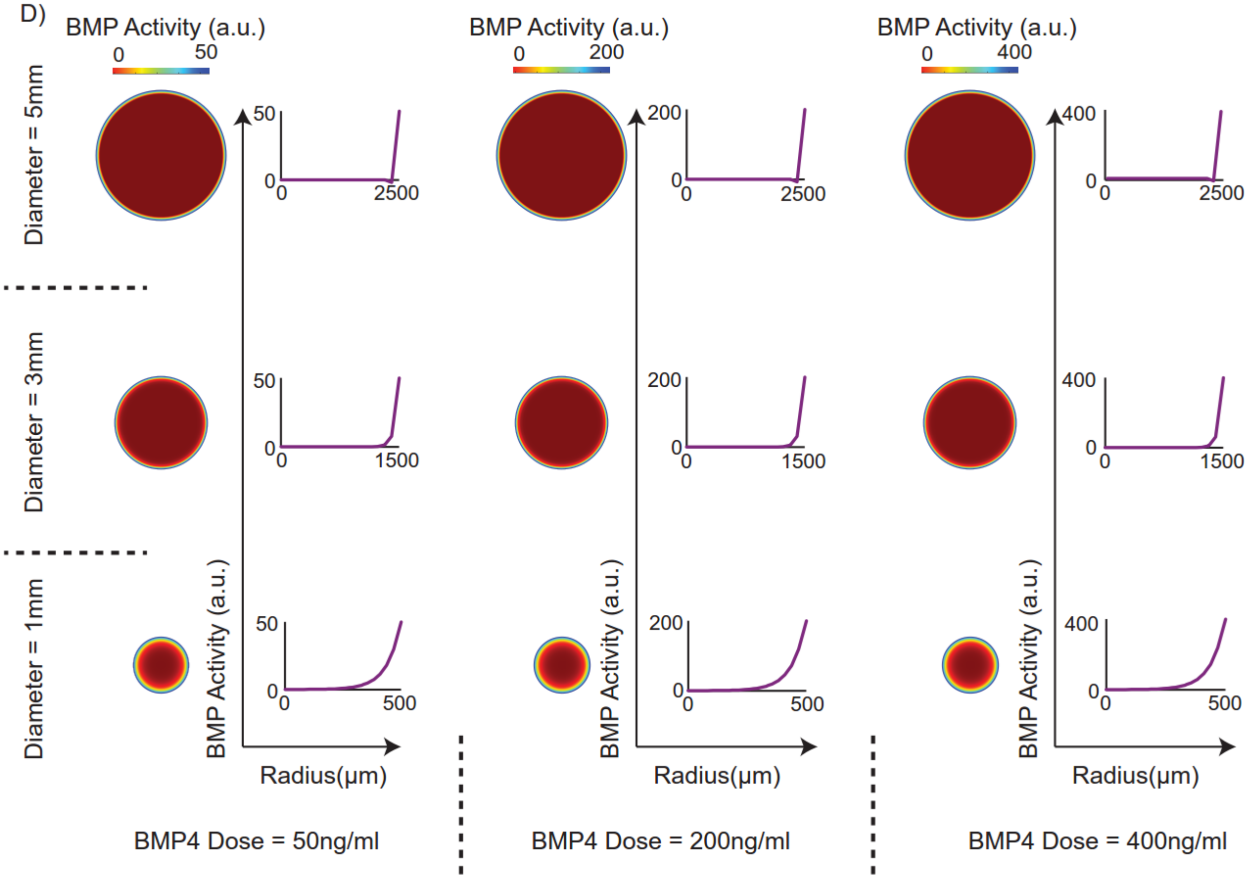

Figure S13: Dual inhibition model does not give rise to repetitive RD-like free BMP4 distribution. A) Proposed dual-inhibition model of gradient formation in differentiating hPSC colonies by Etoc *et al*(Etoc et al. 2016). B) Simplified mathematical representation of a generic dual inhibition model. C) Gradient formation of free BMP4 ligands as predicted by the model. D) Varying doses and colony sizes demonstrates the inability of the dual-inhibition model to generate a periodic Turing-like response in the free BMP4 distribution.

Supplementary Materials and Methods: Model Description

Background of the model

To test the assertion that a BMP4-NOGGIN RD system was self-organizing the pSMAD1 gradient, we set out to develop an RD model specific to our tissue geometry, initial, and boundary conditions to query testable predictions of signaling gradient formation that may arise in our geometrically confined hPSC colonies. Notably, the purpose of this model was not to attain detailed information of the response of the signal transduction within the differentiating cells, or identify the gene regulatory network that would allow differential induction of fates in response to different signaling levels. Instead, we set out to identify the simplest possible RD model that could produce a BMP signaling gradient for a 1000µm diameter colony when differentiated with 50ng/ml of BMP4 in the induction media – reminiscent of the pSMAD1 signaling patterns observed in our experimental data under those specific conditions. We then validated this model based on the predictions to perturbations of two key experimental parameters (**Fig. 4** – main text); and then employed this model to made testable predictions of the differentiation behavior of the hPSC colonies beyond the conditions under which the model was build (**Fig. 6, 7** - main text). The RD model described below is a simplified, and an idealized model. Nevertheless, this model generates predictions of morphogen (BMP4) distribution in response to perturbations of experimental conditions which is constantly shown to be consistent with both pSMAD1 gradient formation and associated hPSC differentiation.

Two-component Reaction-Diffusion system

We set out to develop a mathematical model of the concentration profiles of BMP4 and NOGGIN molecules as a function of space and time in our micro-patterned colonies, based on a reaction-diffusion (RD) system (Turing 1952; Murray 2008; Gierer & Meinhardt 1972). The partial differentiation equation (PDE) set can be described as follows:

$$\frac{\partial bmp}{dt} = F(bmp, nog) - d_{BMP} bmp + D_{BMP} \nabla^2 bmp$$

$$\frac{\partial nog}{dt} = G(bmp, nog) - d_{NOG}nog + D_{NOG}\nabla^2 nog \tag{1}$$

Here, *bmp*, and *nog* are functions of both space, and time that represent the local concentrations of BMP4 and NOGGIN molecules at a particular point in our micro-patterned colonies*. F (bmp, nog)*, and *G (bmp, nog)* represent the non-linear functions which describe the production rates of BMP4, and NOGGIN. The degradation rates of the molecules are given by d_{BMP}, and $d_{NOG;}$ and D_{BMP}, and D_{NOG} represent the diffusivities of the molecules.

We assumed that the production terms of BMP4, and NOGGIN can be approximated by linear functions (close to the steady state). The nature of the production terms is such that as the values of *F*(*bmp,nog*), and *G*(*bmp,nog)* increase, the system transitions away from steady state, preventing convergence of the solutions(Murray 2008; Kondo & Miura 2010). Attempts to restrict the values for the morphogen near steady state, to enable convergence, have either used non-linear functions that saturate at increasing values (e.g. the Hill function(Sick et al. 2006)) or have enforced a range in which the linear approximation of the reaction function is confined(Kondo & Miura 2010). Since we used linear production functions (2) in our model (1), we chose the latter strategy and restricted the reaction functions to a defined range(Kondo & Miura 2010). The production terms are represented by:

$$\begin{aligned} F(bmp, nog) &= 0 \leq a_{BMP}bmp + b_{BMP}nog + c_{BMP} \leq 1 \\ G(bmp, nog) &= 0 \leq a_{NOG}bmp + b_{NOG}nog + c_{NOG} \leq 5 \end{aligned} \tag{2}$$

Changing Variables:

To circumvent the issue of intractability of the number of BMP4 and NOGGIN molecules in the circular region of interest modelled by our PDE solutions, we chose to change the variables *bmp*, and *nog* into normalized, dimensionless variables which we represent as *bmp**, and *nog**.

<u>Quantities, and assumptions of note</u> – *All experiments were performed in micro-patterned 96-well plates with a volume of a **100µl** of induction media per well. The culture surface area of each well is **0.3165cm²**. The molecular weight of BMP4 is **34KDa**. We assumed that the two-dimensional colony 'surface' across which the BMP4 and NOGGIN distributions are predicted in the PDE set could be approximately represented by a **1µm** height from the colony surface. Therefore, the equivalent 'surface concentration' of **one colony of 1mm diameter** when **100ul** of induction media containing **1ng/ml** of BMP4 in SI units is <u>1.77×10^{10} **molecules/m²**</u>. We opted to change the variables (bmp, nog) by normalizing the entire PDE set by 1.77×10^{10} molecules/m².*

The linearized PDE set from (1), and (2) together are of the following form:

$$\frac{\partial bmp}{\partial t} = a_{BMP}bmp - b_{BMP}nog + c_{BMP} - d_{BMP}bmp + D_{BMP}\nabla^2 bmp$$

$$\frac{\partial nog}{\partial t} = a_{NOG}bmp - b_{NOG}nog + c_{NOG} - d_{NOG}nog + D_{NOG}\nabla^2 nog \qquad (3)$$

The SI units for the parameters, and variables in (3) are shown below:

$bmp\ [\frac{molecules}{m^2}]$	$nog\ [\frac{molecules}{m^2}]$
$a_{BMP}\ [\frac{1}{s}]$	$a_{NOG}\ [\frac{1}{s}]$
$b_{BMP}\ [\frac{1}{s}]$	$b_{NOG}\ [\frac{1}{s}]$
$c_{BMP}\ [\frac{molecules}{m^2 \times s}]$	$c_{NOG}\ [\frac{molecules}{m^2 \times s}]$
$d_{BMP}\ [\frac{1}{s}]$	$d_{NOG}\ [\frac{1}{s}]$
$D_{BMP}\ [\frac{m^2}{s}]$	$D_{NOG}\ [\frac{m^2}{s}]$

Dividing throughout by $1.77x10^{10}$ ***molecules/m²***, we changed the variables *bmp, nog,* c_{BMP}, and c_{NOG} as follows:

$bmp\ [\frac{molecules}{m^2}] = bmp^*\ [\ ]\ x1.77x10^{10}\ [\frac{molecules}{m^2}]$	$nog\ [\frac{molecules}{m^2}] = nog^*\ [\ ]\ x1.77x10^{10}\ [\frac{molecules}{m^2}]$
$c_{BMP}[\frac{molecules}{m^2 Xs}] = c^*_{BMP}\ [\frac{1}{s}]\ x1.77x10^{10}\ [\frac{molecules}{m^2}]$	$c_{NOG}[\frac{molecules}{m^2 Xs}] = c^*_{NOG}\ [\frac{1}{s}]\ x1.77x10^{10}\ [\frac{molecules}{m^2}]$

Taken together, the updated set of partial differential equations with the changed variables (*bmp*, nog**, c^*_{BMP}, *and* c^*_{NOG}) is given below:

$$\frac{\partial bmp^*}{\partial t} = a_{BMP}bmp^* + b_{BMP}nog^* + c^*_{BMP} - d_{BMP}bmp^* + D_{BMP}\nabla^2 bmp^*$$

$$\frac{\partial nog^*}{\partial t} = a_{NOG}bmp^* + b_{NOG}nog^* + c^*_{NOG} - d_{NOG}nog^* + D_{BMP}\nabla^2 nog^* \qquad (4)$$

Development • Supplementary information

Dose dependence in BMP4 and NOGGIN production

We observed a BMPi dose dependent production of both BMP4 and NOGGIN (**Fig S5**). To incorporate this response into our model we chose the following expressions for a_{BMP}, and a_{NOG}.

$$a_{BMP} = \alpha(1 + BMPi * \gamma_{BMP})$$
$$a_{NOG} = \alpha(1 + BMPi * \gamma_{NOG}) \quad (6)$$

Initial conditions of BMP4 and NOGGIN distributions in micro-patterned colonies:

BMP4

BMP4 is added in the differentiation medium, and presented to the colonies at a uniform dose. Therefore, we considered the initial concentration of BMP4 to be a constant value given by *BMPi*.

NOGGIN

The initial conditions for NOGGIN are more nuanced. Although NOGGIN is produced in response to BMP signaling in the cardinal 'Activator-Inhibitor' paradigm, BMP4 ***inhibition*** in the initial condition (at time t=0), can be achieved by a variety of different molecules (e.g. FOLLISTATIN (FST), CHORDIN, GDF3, and CERBERUS-Like (CERL) among others) in addition to NOGGIN(Wu & Hill 2009). Since we observed elevated basal expression of BMP signaling inhibitors like FST, GDF3, and CERL relative to NOGGIN in hPSCs during basal culture conditions (**Fig. S4B-G**), we opted to consider the spatial profile of a 'generic BMP inhibitor' as the initial condition for the RD paradigm.

To identify the specific spatial profile of a generic BMP inhibitor, we developed a simplified model of a passive diffusion-driven profile that would arise in a circular hPSC colony where each cell is a source of the secreted molecule. Over the course of the formation of a confluent hPSC colony, we assumed that the expression profile of a 'generic BMP inhibitor' would reach a steady state. To approximate this steady state spatial profile, we considered each cell (a point source of the inhibitor), evenly distributed within the colony (**Fig. S14A**), and assumed an infinite sink at a large distance from the colony (**Fig. S14B**). Simulation of a steady state response revealed a spatial profile that could broadly be approximated as an elliptical paraboloid (**Fig. S14C-D**). Accordingly, we considered the initial condition of NOGGIN, which at t=0 can be replaced by the effective contribution of all BMP inhibitors being expressed by the hPSCs in the micro-patterned colony, to be an elliptical paraboloid function (**Fig. S14E**). Notably the peak concentration chosen for the NOGGIN initial condition is arbitrary – and the patterning of free BMP4 distribution was found to be robust to the choice of the peak value.

Development • Supplementary information

Boundary conditions for the BMP4-NOGGIN reaction-diffusion system in micro-patterned colonies

We assumed that the cells at the radial edge of the micro-patterned colonies were always subjected to the same concentration of BMP4 that is in the bulk medium, which parallels the 'edge-sensing' model that has been proposed by previous studies(Etoc et al. 2016). For the case of NOGGIN, we assumed a no flux boundary condition.

Final Reaction-Diffusion PDE

The final two-component PDE can be written as shown below:

$$\frac{\partial bmp^*}{\partial t} = a_{BMP} bmp^* + b_{BMP} nog^* + c^*{}_{BMP} - d_{BMP} bmp^* + D_{BMP} \nabla^2 bmp^*$$

$$\frac{\partial nog^*}{\partial t} = a_{NOG} bmp^* + b_{NOG} nog^* + c^*{}_{NOG} - d_{NOG} nog^* + D_{BMP} \nabla^2 nog^*$$

Initial Conditions

bmp(t=0) = BMPi*

nog(t=0)* = $\{0 \le -\left(\frac{x}{R}\right)^2 - \left(\frac{y}{R}\right)^2 + 1\}$

Boundary Conditions

bmp(boundary) = BMPi*

$$\frac{d(nog * (boundary))}{dt} = 0$$

In the initial condition for Noggin, *R* represents the colony radius.

Development • Supplementary information

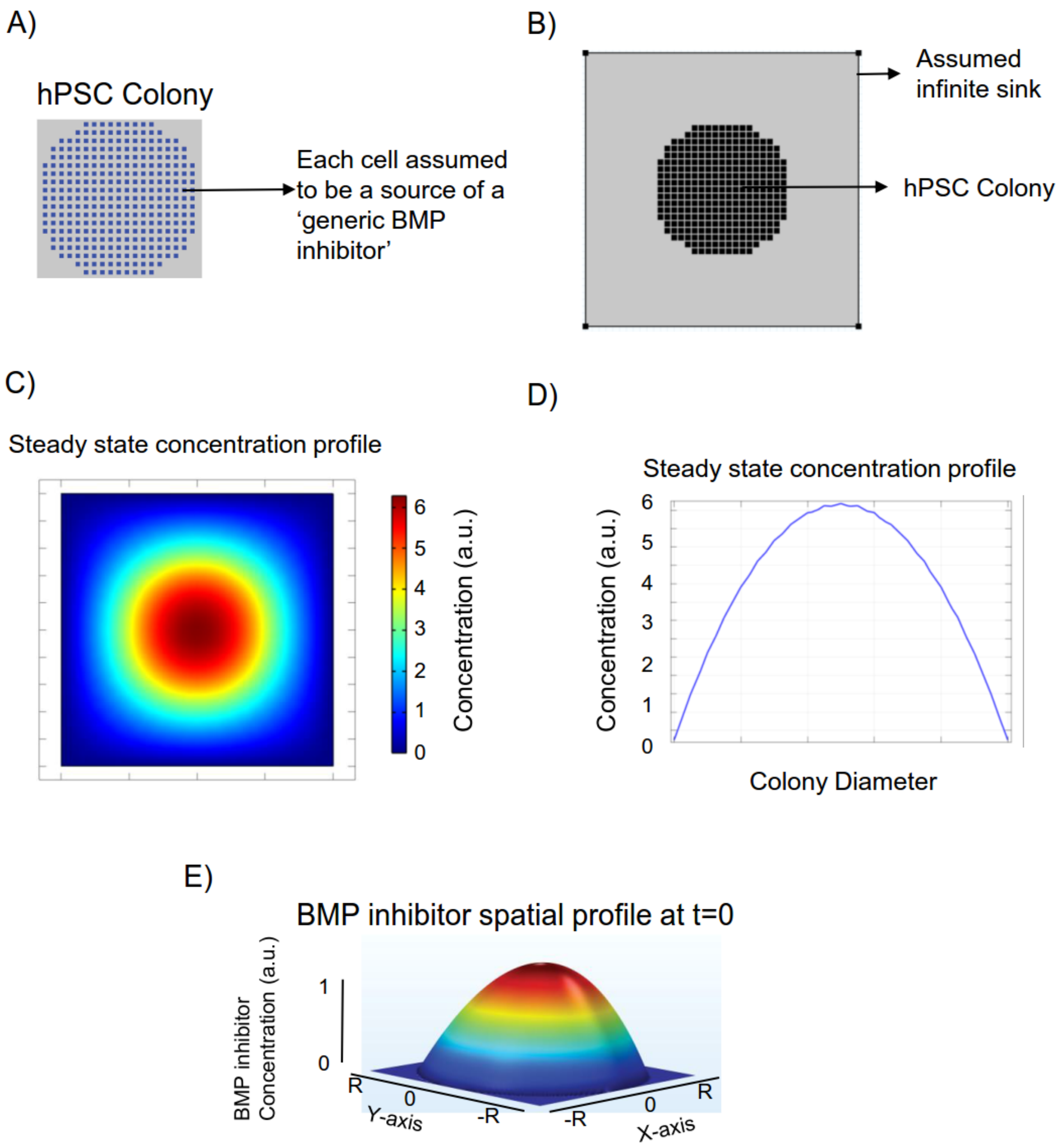

Figure S14: Initial condition for Noggin. A) A circular colony of cells (blue dots) modeled as a collection of sources of secreted molecules. B) Noggin is assumed to have an infinite sink at a certain distance from the colony periphery. The steady state diffusion profile of the secreted molecule is shown in C), and the expression profile within the colony along the diameter shown as a line plot D). E) The assumed initial condition of Noggin at the start of induction. 'R' represents the colony radius.

Development • Supplementary information

Parameter choices and parameter sensitivity

Importantly, the coefficients ka_{BMP}, b_{BMP}, c^*_{BMP}, ka_{NOG}, b_{NOG}, and c^*_{NOG} in the production terms for BMP4, and NOGGIN do not correspond to experimentally determined parameter values. The values for these parameters were chosen as per Kondo et al(Kondo & Miura 2010) – we first chose a parameter set that resulted in oscillating values of BMP4, and NOGGIN, and then chose the values of the diffusion coefficients such that $D_{NOG} > D_{BMP4}$ (**Table S3**).

The diffusivity values for both NOGGIN, and BMP4 are in realistic ranges (Raspopovic et al. 2014; Sick et al. 2006; Inomata et al. 2013). For instance, Inomata et al. calculated the diffusivity of NOGGIN in Xenopus embryos to be 37±6.6 μm^2/s, which is close to the predicted diffusivity of Noggin in our system. However, the exact values of the diffusivities in our system have not been measured.

The value for b_{NOG} was chosen to be zero since NOGGIN does not have any receptors and is therefore, unable to repress its own expression.

Table S3: Model parameters

**Parameter**	**Value**	**Adapted from**
α	0.005 [1/s]	Kondo et al.
b_{BMP}	0.01[1/s]	Kondo et al.
c^*_{BMP}	0.003 [1/s]	Kondo et al.
d_{BMP}	0.003 [1/s]	Kondo et al.
D_{BMP}	11 [μm2/s]	-
γ_{NOG}	0.0025	Kondo et al.
b_{NOG}	0	-
c^*_{NOG}	-0.015 [1/s]	Kondo et al.
d_{NOG}	0.009 [1s]	Kondo et al.
D_{NOG}	55 [μm2/s]	-

In **Fig. S15**, we show the response of the predicted spatial profile of free BMP4 molecules within the geometrically-confined hPSC colony to varying the above parameters to provide a sense of the sensitivity of the model output to the model parameters. The sensitivity of the model to perturbing the mesh definition is shown in **Fig. S16**.

Development • Supplementary information

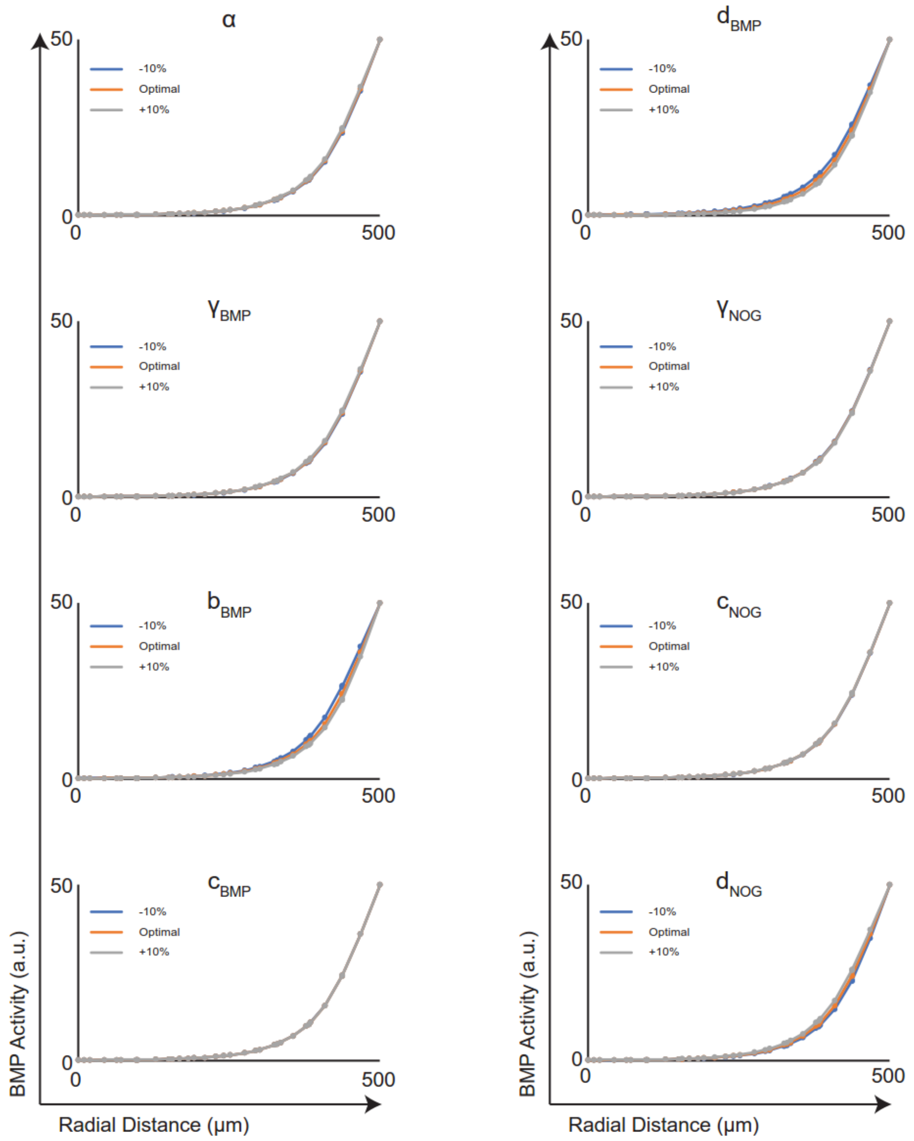

Figure S15: *Response of predicted gradient formation to perturbation of model parameters.*

Development • Supplementary information

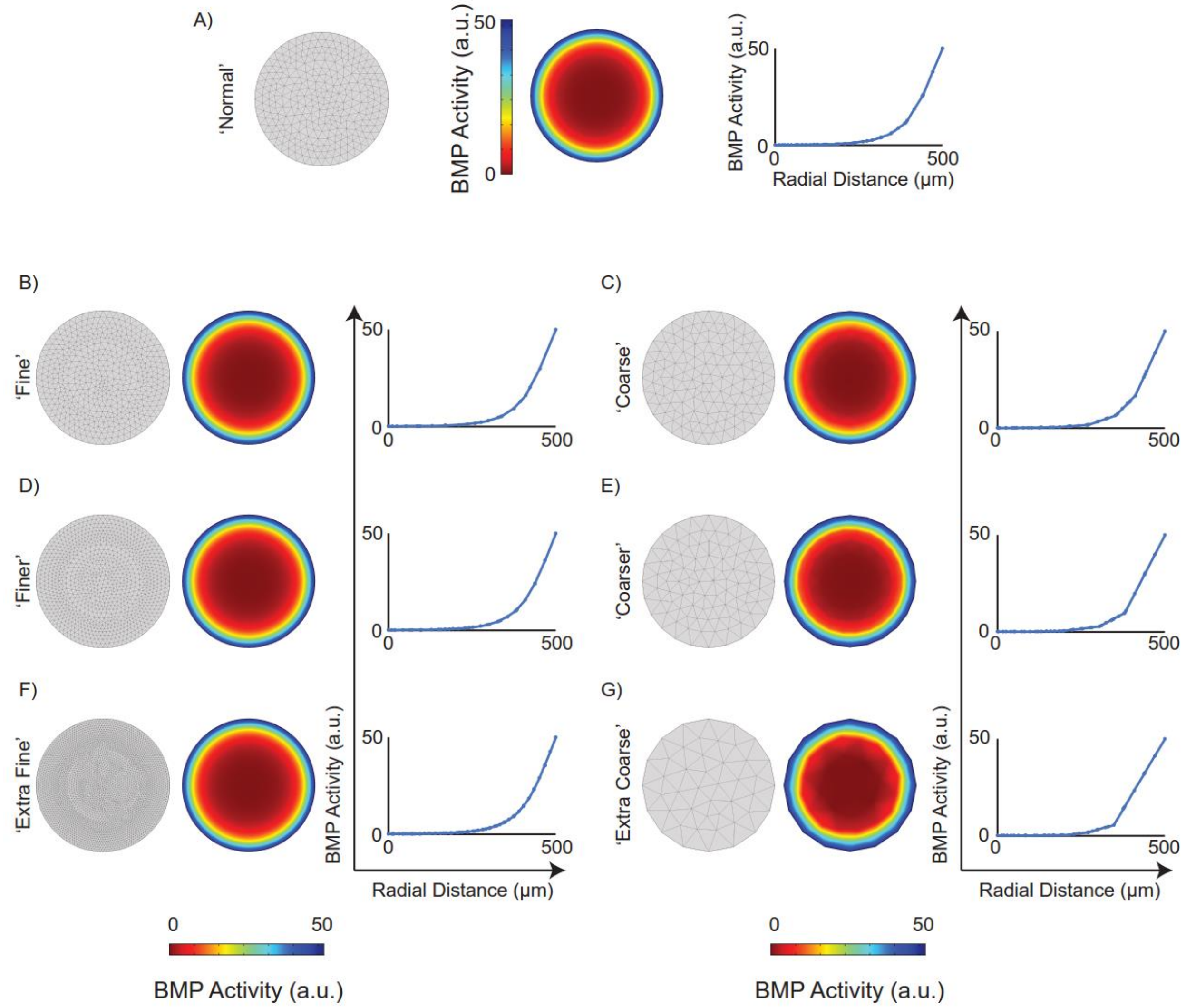

Figure S16: *Gradient formation predicted with pre-defined mesh sizes in Comsol A) 'Normal', B) 'Fine', C) 'Coarse', D) 'Finer', E) 'Coarser', F) 'Extra Fine', and G) 'Extra Coarse'.*

References:

Etoc, F., Metzger, J., Ruzo, A., Kirst, C., Yoney, A., Ozair, M.Z., Brivanlou, A.H. & Siggia, E.D., 2016. A Balance between Secreted Inhibitors and Edge Sensing Controls Gastruloid Self-Organization. *Developmental Cell*, 39(3), pp.302–315.

Gierer, A. & Meinhardt, H., 1972. A theory of biological pattern formation. *Kybernetik*, 12(1),

Development • Supplementary information

pp.30–39.

Inomata, H., Shibata, T., Haraguchi, T. & Sasai, Y., 2013. Scaling of Dorsal-Ventral Patterning by Embryo Size-Dependent Degradation of Spemann's Organizer Signals. *Cell*, 153(6), pp.1296–1311.

Kondo, S. & Miura, T., 2010. Reaction-Diffusion Model as a Framework for Understanding Biological Pattern Formation. *Science*, 329(5999), pp.1616–1620.

Murray, J.D., 2008. *Mathematical Biology II - Spatial Models and Biomedical Applications* Third. Springer, ed.,

Raspopovic, J., Marcon, L., Russo, L. & Sharpe, J., 2014. Digit patterning is controlled by a Bmp-Sox9-Wnt Turing network modulated by morphogen gradients. *Science*, 345(6196), pp.566–570.

Sick, S., Reinker, S., Timmer, J. & Schlake, T., 2006. WNT and DKK Determine Hair Follicle Spacing through a Reaction-Diffusion Mechanism. *Science*, 314(5804), pp.1447–1450.

Turing, A., 1952. The chemical basis of morphogenesis. *Bulletin of Mathematical Biology*, 237(641), pp.153–197.

Wu, M.Y. & Hill, C.S., 2009. TGF-b Superfamily Signaling in Embryonic Development and Homeostasis. *Developmental Cell*, 16(3), pp.329–343.

Zimmerman, L.B., De Jesús-Escobar, J.M. & Harland, R.M., 1996. The Spemann organizer signal noggin binds and inactivates bone morphogenetic protein 4. *Cell*, 86(4), pp.599–606.

Development • Supplementary information
